# Risk assessment, disease prevention and personalised treatments in breast cancer: is clinically qualified integrative approach in the horizon?

**DOI:** 10.1186/1878-5085-4-6

**Published:** 2013-02-19

**Authors:** Olga Golubnitschaja, Kristina Yeghiazaryan, Vincenzo Costigliola, Daniela Trog, Michael Braun, Manuel Debald, Walther Kuhn, Hans H Schild

**Affiliations:** 1 Department of Radiology, Rheinische Friedrich-Wilhelms-University of Bonn, Sigmund-Freud-Str. 25, Bonn, 53105, Germany; 2 Breast Cancer Research Centre, University of Bonn, Bonn, Germany; 3 European Medical Association, Brussels, Belgium; 4 Department of Obstetrics and Gynaecology, University of Bonn, Bonn, Germany; 5 Department of Gynaecology, Red Cross Clinics Munich, Munich, Germany

**Keywords:** Inflammation, Cancer, Metastasis, Biomarker pattern, Predictive diagnosis, Preventive healthcare, Medical services, Medical record, Integrative personalised medicine, Innovative technologies, Genetic testing, Assay, Omics, Imaging, Immune system, Metalloproteinase, Adjuvant therapy, Computer assistance, Mathematical modelling, Tamoxifen, Ethics

## Abstract

Breast cancer is a multifactorial disease. A spectrum of internal and external factors contributes to the disease promotion such as a genetic predisposition, chronic inflammatory processes, exposure to toxic compounds, abundant stress factors, a shift-worker job, etc. The cumulative effects lead to high incidence of breast cancer in populations worldwide. Breast cancer in the USA is currently registered with the highest incidence rates amongst all cancer related patient cohorts. Currently applied diagnostic approaches are frequently unable to recognise early stages in tumour development that impairs individual outcomes. Early diagnosis has been demonstrated to be highly beneficial for significantly enhanced therapy efficacy and possibly full recovery. Actual paper shows that the elaboration of an integrative diagnostic approach combining several levels of examinations creates a robust platform for the reliable risk assessment, targeted preventive measures and more effective treatments tailored to the person in the overall task of breast cancer management. The levels of examinations are proposed, and innovative technological approaches are described in the paper. The absolute necessity to create individual patient profiles and extended medical records is justified for the utilising by routine medical services. Expert recommendations are provided to promote further developments in the field.

## Review

### Cancer context

With the respect to the statistical data presented by the World Health Organisation [1], cancer is a leading cause of death worldwide, accounting for 7.6 million deaths (around 13% of all deaths) as registered in 2008 and permanently increasing over 13 million as projected for 2030. Economic factors play a role, since about 70% of all cancer deaths in 2008 occurred in low- and middle-income countries. The most fatal types of cancer are listed below in the decreasing order (deaths per year):

❖ lung (1.37 million deaths)

❖ stomach (736 000 deaths)

❖ liver (695 000 deaths)

❖ colorectal (608 000 deaths)

❖ breast (458 000 deaths)

❖ cervical cancer (275 000 deaths).

### Breast cancer is the most common cause of cancer-related death among women

**Figure 1:**
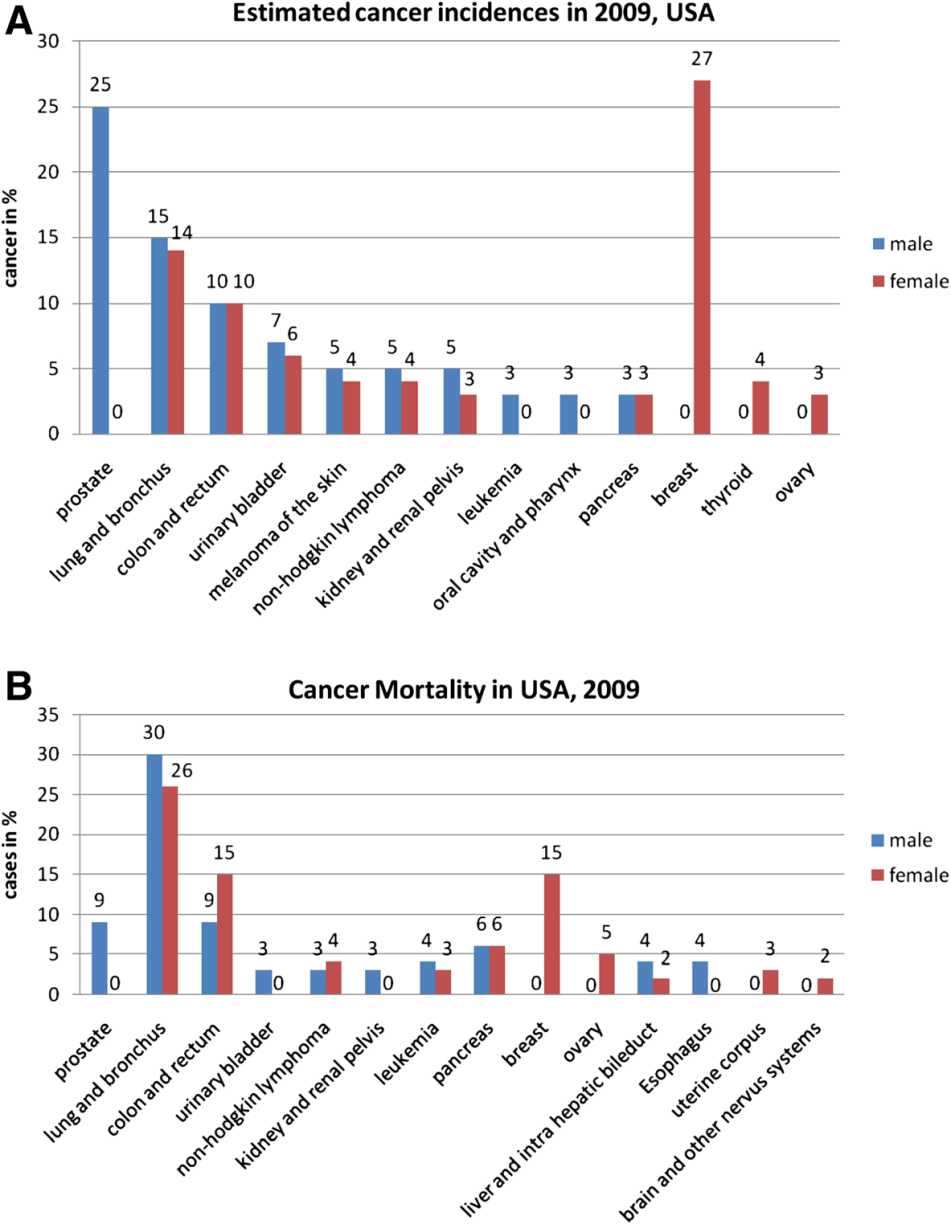
**A. Estimated cancer incidence in USA in 2009; B. Cancer related mortality as registered in USA in 2009; data adapted from **[2]**.**

Hence in the USA, the highest cancer related incidence rates are currently registered for the breast cancer patient cohorts [2] – see Figure 1A. The combating and treating measures such as induced population screening by mammography and application of adjuvant therapies, keep breast cancer mortality mostly unchanged or even persistently declined over last ten years – see Figure 1B. However, the incidence of breast cancer continually increases worldwide during the past three decades. According to the statistical data published by the National Cancer Institute in the USA [3], the estimated new cases and deaths from breast cancer in the United States in 2012 are (in thousand cases)

❖ New cases: 226.870 (female); 2.190 (male)

❖ Deaths: 39.510 (female); 410 (male)

### Breast Cancer Metastatic Disease (BCMD) is currently incurable: challenges of diagnostics and treatment

#### Breast Cancer Metastatic Disease (BCMD)

Diagnostic approaches routinely applied in medical practice are frequently unable to recognise early stages in breast cancer development that impair the outcome. At the time of diagnosis, a great portion of patients with breast cancer have locally advanced and/or distant metastatic disease. It is estimated that about 6% of breast cancer patients demonstrate a clinical picture of metastatic disease already at the time of diagnosis. Further 20% to 50% patients with primary breast cancer will develop metastatic disease despite the standardised treatments approached [4]. BCMD (stage IV) is the most advanced form of breast cancer. Once breast cancer has turned metastatic, the disease is recognised as the incurable one: the 5-year survival barrier will be reached by only 26% of patients treated for the BCMD.

#### Distant metastases

The lion’s share of about 90% of deaths in the overall breast cancer related mortality is caused by the distant metastases. Breast cancer spreads metastasis predominantly into lymph nodes, bone, lung, skin, brain, and liver [5], wherefrom only lymph nodes are considered as non-distance metastases. With the poorest prognosis of approximately 80% mortality rate within first 12 months of diagnosis, brain metastases represent a devastating category of BCMD. Brain metastases are prevalent in hormone receptor negative but HER2-overexpressing subgroups and are typical for 30% of all HER2+ BCMD [4]. The particular challenge in treating brain metastases is created by the limited permeability of the blood–brain barrier for chemotherapeutics, the use of which, further, leads to brain inflammatory response with extensive gliosis surrounding the metastases. The treated brain metastases are further provoked for high proliferation but minimal apoptosis demonstrating unsatisfactory effects of current treatments. Therefore, innovative diagnostic approaches to trace the micrometastases and therapeutic approaches aimed at stabilising and eliminating distant metastases – both do not exist yet being emergent in the nearest future.

#### Diagnosis of BCMD

Advanced imaging technologies are currently considered as being the most appropriate tool to diagnose BCMD, to detect the primary lesions and to trace the distance metastases over the whole body (whole-body imaging). To currently well recognised technologies belong multi-dimensional and multimodal ones: CT, MRI, PET, SPECT, and ultrasound; PET and the combined PET/CT is the key tool for the whole-body scanning. However, there are some substantial clinical deficits which imaging technologies suffer from in pinpointing the disease type [4].

##### RT-PCR

Small-size metastases in lymph nodes may be detected by amplification of the smallest amounts of transcripts produced by BCMD biomarkers such as CK19 and others. The greatest limitation of the methodology is false-positive results potentially received due to the mixed cell populations which cannot be completely excluded by the resection. A conclusion might be also doubtful, due to untargeted biomarkers, particularly for heterogeneous tumours that is, indeed, the frequent case [4].

##### Disseminated and circulating tumour cells

Individual tumour cells in bone marrow and blood stream cannot be detected by conventional imaging. For poor prognosis, more relevant and better detectable are tumour cells disseminated in bone marrow (DTC), compared to circulating tumour cells (CTC) in peripheral blood [6]. However, the invasiveness of the DTC sampling hardly finds the acceptance by patients. Consequently, blood tests for the CTC detection is a promising approach, in particular for the diagnosing of BCMD which demonstrates the most abundant representation of tumour cells in blood followed by high rates of CTC in prostate cancer, in contrast to significantly lower levels of CTC spread by other tumour types [7]. However, this approach suffers from substantial technological limitations such as an extremely low frequency of CTC in a blood stream that makes the tool almost useless for the detection of BCMD at its early stages [8]. Consequently, the reliable results’ interpretation is currently possible only for the advanced stages of the tumour progression / BCMD and for patients with poor prognosis [9]. The promising diagnostic approach might be the molecular characterisation of CTC as the predictor of the tumour invasiveness and therapy response [6].

#### Treatment of BCMD

Currently applied strategies for the treatment of BCMD make use of systemic cytotoxic agents that lead to severe and irreversible organic side-effects significantly decreasing the life quality of the patients followed by a limited long-term success in metastasis suppression: only 1-3% of patients remain long-term disease-free after BCMD treatments [4]. Although new agents like paclitaxel, trastuzumab and aromatase inhibitors improve the short-term survival rates (up to 36 months), the therapeutic goals remain at the level of survival prolongation and symptoms palliation.

The experts are fully consent with the fact that novel drug targets should be elaborated for a successful BCMD treatment tailored to the patient. In this context, molecular defects driving clinical onset of BCMD, beginning with the initiation step to the micrometastasis progression till BCMD virulence, create the robust panel of the drug target candidates [10]. Recent reports from animal models of BCMD treatments keep a hope in potential improvements which, however, are not going to happen for the patients tomorrow.

### Breast cancer risk assessment

#### “Molecular portrait“ and more

Early detection of the tumour has been demonstrated to be highly beneficial for significantly enhanced therapy efficacy. An accurate navigation by predictive diagnosis may lead to full recovery after surgical resection [11]. Furthermore, a detection of individual predisposition to breast cancer represents the optimal way how the pathology may be diagnosed before its clinical onset and development of the fatal BCMD. Breast cancer risk assessment is currently extensively under consideration. The major problem, however, is linked to the multifactorial nature of the disease. Consequently, the list of parameters with impacts for the disease onset and progression at the individual level, i.e. personal risk factors differ significantly from patient to patient. This consideration leads to better understanding, why the “across-the-board” treatment of breast cancer is frequently ineffective, and the pathology specific “portrait” should be created at the individual level. On this, any biological manifestation is operated and controlled at the molecular level. Therefore, the “portrait featuring” originates from the specific set-up of individual biomolecules and corresponding interaction among relevant pathways at molecular, subcellular and cellular levels. This “molecular portrait” creates an individual condition for the disease predisposition and promotion, which is recognisable and modifiable through individual pathology specific “molecular patterns”. For the clinically relevant and issue sensitive interpretation, the informational input from the “molecular patterns” should be combined with complementary technologies such as medical imaging, which altogether contribute to the creation of the individual “patient profiles” as the robust platform for personalised healthcare services. The expected outcomes are conducive to more effective population screening, prevention early in childhood, identification of persons at-risk, stratification of patients for the optimal therapy planning, prediction and reduction of adverse drug-drug or drug-disease interactions.

### Innate immune system as a putative origin of mammary gland

Resulting from the accumulated data from knowledge about morphological particularities, cell composition bioinformatics research, a new concept to the evolutionary origin of mammary gland has been presented suggesting that the gland’s initial function was the provision of innate immunity later evolving into its current nutritional role [12]. Indeed, immune cells are abundant in both physiologic and pathologic mammary tissue. The immune cells are implicated in the development of human mammary glands: leucocytic infiltrates have been detected in normal pubertal and adult gland tissue [12,13]. Furthermore, bone marrow depletion leads to blocked ductal elongation in murine experimental models of mammary gland development. Taking together the above listed facts, the decisive role of the immune cells in physiology of mammary glands is getting obvious. This fascinating discovery opens great perspectives for innovative diagnostic tools based on a minimally invasive blood test platform and might be highly beneficial for novel drug targets of increased efficacy in breast cancer treatments.

### Immune cells and inflammation as tumour modifiers in breast: expression patterns of activated leucocytes collaborative with neoplastic cells under chronic inflammatory condition?

The paradoxical role of leucocytes as protectors, regulators, modifiers and causal players in the breast carcinogenesis becomes extensively discussed in current literatures. Both innate (myeloid) and adaptive (lymphoid) leucocyte types have been demonstrated as breast cancer modifiers [14]. Doubtless cytotoxic T-lymphocytes have a function in constraining tumour developments that is evident, in particular, for the tumours of viral origin [15]. On the other side, the chronic activation of leucocytes paradoxically play a role in initiating / potentiating carcinogenesis: infiltrating B-lymphocytes have been reported to represent the predominant lymphocytic population in premalignant breast tissue [14]. Further, B-cells represent the predominant lymphocytes during early breast cancer, whereas infiltrating T-lymphocytes are more extensive in higher graded ductal *in situ* and invasive breast carcinomas [16,17].

What is the mechanism of the tumour promotion by inflammatory leucocytes? The key-point is their unique plasticity in producing protein products and bioactive mediators essential for all stages in the tumour progression such as reactive oxygen species, tissue-remodelling (e.g. metalloproteinases) angiogenesis prompting (e.g. VEGF) protein-complexes [18,19]. Certainly, this enormous capacity is conditioned by the stage specific expression patterns in activated leucocytes. Under the chronic inflammatory condition the expression patterns of infiltrating leucocytes obviously become collaborative with those of neoplastic cells. An excellent example is provided by tissue-remodelling proteins secreted from activated leucocytes. An altered metalloproteinase activity impacts directly the mammary gland physiology during morphogenesis, hormonal cycle and lactation, as well as during inflammatory acute / chronic process, cancer pre-lesions, tumour progression, and metastatic disease. Besides other cell types in the population, inflammatory and immune cells are the major producers of metalloproteinases [20]. Although the impacts of the metalloproteinase activities are well acknowledged for mammary glands physiology and pathophysiology, the relevance of the metalloproteinase patterns as the breast cancer modifiers in the context of inflammation and immune cells represents won its recognition only recently in the scientific world [21].

### Molecular patterns in activated leucocytes as the minimally invasive diagnostic tool for breast cancer risk assessment

Pursuing the above conclusions, it is getting obvious that the molecular/expressional patterns in orchestrated leucocytes are activated strictly in accordance to the precancerous / cancer stage. If detected in correlation with the corresponding disease initiation and progression stage, these patterns in activated leucocytes might be of high relevance for the diagnostic and treatment purposes. This consideration leads to the idea of creating a minimally invasive approach for breast cancer risk assessment based on *ex vivo* blood tests by examination of the specific molecular/expressional patterns in circulating leucocytes.

### The OVERALL TASK: Multimodal diagnostic approaches, disease specific biomarker-patterns, individual patient profiles, creation of medical records and treatments tailored to the person

Paradigm change from a delayed approach after clinical onset of the pathology to predictive diagnostics followed by targeted prevention and individualised treatment algorithms tailored to the patient, creates an innovative concept for advanced healthcare that is costs effective [22]. Particularly attractive are non-invasive diagnostic approaches considering disease-specific alterations in molecular patterns of blood cells and serum in predisposed individuals before clinically disease onset [11,23-29]. Identification of pathology-specific biomarker-patterns increases the specificity and predictive power of analytical approach. Combination of patterns at subcellular, intracellular and extracellular levels contributes to high sensitivity and specificity of the analysis. Mathematic modelling of patient-specific profiles allows for an accurate prediction of individual predisposition before the pathology is manifested. Integrative medical approach by predictive diagnostics, targeted prevention and personalised treatments is considered as the medicine of the future. The expected outcomes are conducive to more effective population screening, prevention early in life, identification of persons at-risk, stratification of patients for the optimal therapy planning, prediction and reduction of adverse drug-drug or drug-disease interactions relying on emerging technologies, such as medical imaging, pharmacogenetics, *omics, disease modelling, individual patient profiles, integrative medical records, etc.

### Technological design: integrative concept

**Figure 2:**
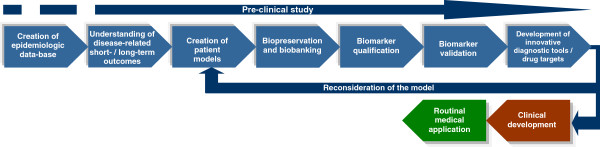
Moving from basic research to clinical implementation: basic steps in creating the robust diagnostic platform and treatments tailored to the person.

The integrative concept of the technological design is summarised in Figure 2. An optimal sep-up of stakeholders and a high quality of the performance of single operating steps (sub-projects) guarantee for a discovery and qualification of innovative diagnostic approaches and valid drug targets to be successfully implemented in clinical practice. The crucial step in the overall experimental scheme is a well-established patient model that reflects the clinical condition(s). Large-scaled studies to identify novel diagnostic biomarkers and therapeutic targets followed by validation, standardisation and application procedures are essential in breast cancer research.

### Creation of medical records

Creation of medical records is the crucial step in the overall task of prediction, precise disease diagnosing and successful application of the treatment algorithms tailored to the person. Medical record should carry an integrative character presenting and evaluating disease relevant data at any applicable level of the examination / detection. The major points to be obligatory involved in the medical records related to the breast cancer are summarised below:

Sur/name

Date of birth / Age

Ethnicity [30]

Menopausal status [30]

Menstrual cycle (duration, regularity etc.)

History of pregnancies and childbirth

Last date, type and result of past individual cancer screening (mammography, pap smear etc.)

Breast / Cancer familial background (as described elsewhere)

Histological statement for malignant tumours / benign indication

Drug history: alcohol, nicotine etc.

Medication history (i.e. steroids, blood pressure medication, anti-inflammatory medication etc.)

For malignant tumours: evaluation of combined results by medical imaging, categorisation of the carcinoma (invasive lobular, ductal carcinoma *in situ*, etc.), TNM staging (size of cancer, nodal status, type of metastases, receptor status, HER2, etc.) molecular subtypes (luminal a & b, basal, etc.)

For benign patients: acknowledged breast cancer risk factors (childless, lack of breast feeding, breast trauma / inflammations / biopsy, etc.) [30]

Frequent co-morbidities (Diabetes type 2, cardiovascular disease, depression) [31,32]

Environmental particularities (geographic factors, environmental toxicity, such as an excess of heavy metals and toxic compounds as described elsewhere)

Inactive life-style and overweight (body mass index) that influence the pathology development and outcomes [31,32]

Sleep disorders as the predisposition and the cause of cancer [33]

Detectable stress factors with acknowledged impacts for BC development such as a shift-worker’s job [34]

Breast / Cancer specific molecular patterns in blood (as discussed later in text)

Metastasis specific biomarkers in blood (medical imaging and CTC detection as discussed above)

### Construction of diagnostic windows for minimally invasive breast cancer risk assessment based on immune cells profiling

This multimodal approach utilises a combination of conventional analytical methodology for a creation of the pathology specific biomarker patterns at complementary levels of detection, namelyfollowed by mathematical modelling of pathology-specific profiles.

•Medical imaging (primary tumour, distanced metastasis)

•Subcellular / molecular imaging by “comet assay” DNA analysis (risk assessment for general tumour predisposition)

•Clinical differential proteomics as the “gene hunting” approach for pathology specific molecular patterns in blood cells

•Blood metabolomics for quantification of disease relevant metabolite patterns

•Quantitative analysis of enzymatic activities in blood plasma

•others

Here we demonstrate the analytical procedure for two levels of detection, namely molecular imaging by quantitative “comet assay” and clinical proteomics.

### Subcellular / molecular imaging by “comet assay”-analysis

**Figure 3:**
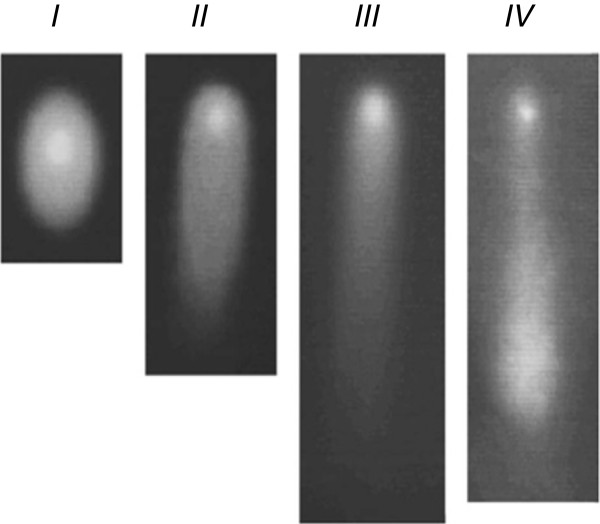
**Image of the characteristic classes of comets (representing intact and damaged DNA) are shown *ex vivo *for circulating leucocytes **[35]**.**

The “comet assay” provides a simple and effective method for evaluation of DNA damage and DNA-repair capacity in single cells such as leucocytes. The principle of the assay is based upon the ability of DNA fragments to migrate out of the cell under the influence of an electric field. An evaluation of the “comet” tail shape and DNA fragments migration pattern allows for assessment of DNA damage and repair capacity. DNA-damage is assigned to 4 classes based on the visual aspect of the comets, considering the extent of DNA migration as published earlier [35]. Comets with a bright head and almost no tail are classified as class I with minimal DNA damage. Comets with no visible head and a long diffuse tail are classified as class IV (severely damaged/apoptotic cells). Comets with intermediate characteristics are assigned to classes II and III dependent on the ratio R = T/r, where T is a length of comet´ s tail and r is a radius of comet´ s head. The characteristic value of R for class 1 is 1 (T ≈ r) and for class 4 is ∞ (r = 0). Comets with values 1<R<3 are classified as class 2 (see the original image). Comet classes are demonstrated with the image provided in the Figure 3.

Subcellular / molecular imaging by quantitative “comet assay” has characterised the breast cancer patients as follows:

➢ Increased damage to DNA

➢ Debilitated apoptotic reaction towards increased DNA damage

➢ Pathology specific comet patterns

➢ Impact of hormonal status on specificity of comet patterns among breast cancer patients

➢ Characteristic windows of comet patterns that may be utilised for breast cancer risk assessment – both positive (at high-risk) and negative (at low-risk) prediction.

**Figure 4:**
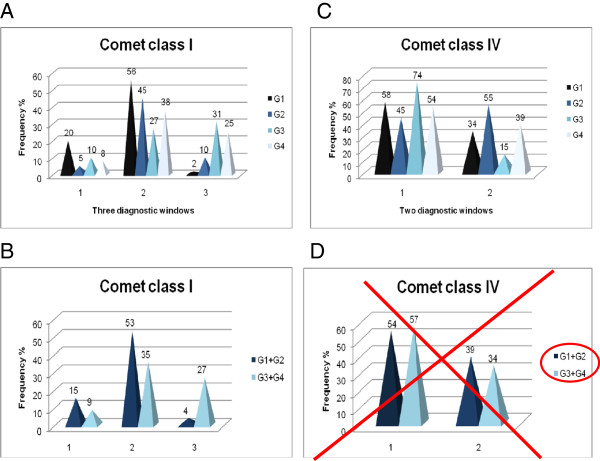
**Diagrams estimating a predictive power of the comet-fractions (comet class I and IV), further utilised in the construction of diagnostic windows for breast cancer risk assessment (A, B and C); according to the diagnosis, the recruited patients are grouped as follows: pre-menopausal women with benign alterations in breast tissue (G1); post-menopausal women with benign alterations in breast tissue (G2); invasive lobular & ductal carcinomas in pre-menopausal women (G3); invasive lobular & ductal carcinomas in post-menopausal women (G4); data taken from **[36]**.** Obviously, the diagnostic windows with the comet class IV patterns can be effective only when the hormonal status is considered as one of the selection parameters for subgrouping the patients and concomitant utilisation of the analytical approach proposed by this study.

An example of the diagnostic windows for breast cancer risk assessment using comet classes I (intact DNA) and IV (apoptotic) is demonstrated in Figure 4[36]. The constructed diagnostic windows clearly distinguish between tumour and benign patients and may be considered for the practical application in differential molecular diagnostics. For this diagnostic tool two parameters in medical records are of particular importance, namely the age and menopausal status.

### Clinical differential proteomics as the promissing tool for breast cancer risk assessment

#### Protein mapping in circulating leucocytes of breast cancer patients

**Figure 5:**
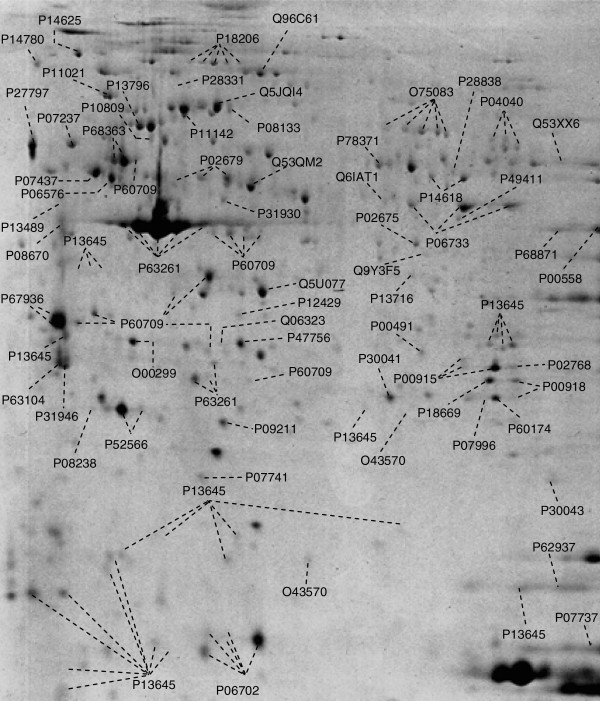
**Protein mapping in circulating leucocytes of breast cancer patients; first-dimensional separation was performed in immobilised pH gradient (IPG) strips (Bio-Rad, USA) in the range of IP 4–7.** Following first-dimensional separation, the extruded IPG-strips were equilibrated in gel equilibration buffer I (50 mM Tris–HCl, 6 M urea, 30% glycerol, 2% SDS, 1% DTT), followed by equilibration in buffer II (50 mM Tris–HCl, 6 M urea, 30% glycerol, 2% SDS and 260 mM iodacetamide) for 10 min before loading them onto polyacrylamide gels (12% SDS-PAGE) for the second-dimensional resolution in Mini-PROTEAN 3 (Bio-Rad). Altogether, 74 proteins were consequently identified by MALDI-TOF analysis; data taken from [11].

The protein mapping performed in our recent project resulted in altogether 158 protein spots distinguished; the overall spots correspond to 74 proteins the amino acid sequences of which have been consequently identified utilising the analytical technology of MALDI-TOF – see Figure 5[11]. The identified proteins are listed in the Table 1.

**Table 1:** Protein profile alterations in breast cancer and under radiotherapy

**Spot number**	**Access number**	**Accession name**	**Protein name**	**Functional group number**	**Classification, references relevant for functional groups 19, 20 and 21**	**Profile alterations *versus *controls**	**Alterations under radio-therapy**
**CATEGORY A: significantly (T≤0.1) altered expression profiles in patients *versus *controls**							
**112-116**	**P04040**	CATA_HUMAN	Catalase	5, 9, 10, 11, 14, 18, **19, 20, 21**	anti-oxidant defence and detoxification protein [37-42]	**homogeneous suppression **⬇ **3x T=0,001**	Individual reaction ⬆ ⬇
**157**	**P07737**	PROF1_HUMAN	Profilin-1	1, 2, 11, **19, 20, 21**	Microfilamental network cell-migration related protein [11,43-48]	**homogeneous upregulation **⬆ **4,0x T=0,02**	**homogeneous suppression **⬇ **T=0,05**
**23-27**	**P63261**	ACTG_HUMAN	Actin, cytoplasmic 2 (Gamma-actin)	1, 2, 11, 14, 18, **19, 20, 21**	Microfilamental network protein [49-52]	**homogeneous suppression** ⬇ **2x T=0,02**	Individual reaction ⬆ ⬇
**124**	**P27797**	CRTC_HUMAN	Calreticulin precursor CRP55	2, 11, 12, 17, 18, **19, 20, 21**	Endoplasmic reticulum calcium-storage protein regulating focal adhesion and cell motility [53-60]	**homogeneous suppression** ⬇ **2x T=0,02**	Individual reaction ⬆ ➜ ⬇
**155**	**P30043**	BLVRB_HUMAN	Flavin reductase, NADHP-dependent reductase	3, 6, 9, 11, 18, **19**	Riboflavin biosynthesis pathway [61]	**individual induction **⬆ **T=0,02**	**homogeneous induction **⬆ **T=0,002**
**70-74**	**P13645**	K1C10_HUMAN	Keratin, type I cytoskeletal 10	1, 2, 11, 18, **19, 20, 21**	Microfilamental network protein [62-67]	**homogeneous induction **⬆ **T=0,03**	**homogeneous suppression** ⬇ **T=0,1**
**136**	**O00299**	CLIC1_HUMAN	Chloride intracellular channel protein 1	8, 11, 14, **19, 20, 21**	Channel, osmosis, Ca^2+^-dependent apoptosis-related protein [68-71]	⬇ **2,5x T=0,04**	Individual reaction ⬆ ⬇
**156**	**P08238**	HS90B_HUMAN	Heat shock protein HSP 90-beta	12, 13, 14, 11, 17, 18, **19, 20, 21**	Stress response protein [72-76]	**homogeneous suppression** ⬇ **5x T=0,06**	**homogeneous induction **⬆ **T=0,02**
**141**	**P13489**	RINI_HUMAN	Placental ribonuclease inhibitor	3, 9, 12, 14, 17, **20, 21**	RNA/nucleotide turnover pathway [77-83]	**homogeneous suppression** ⬇ **3x T=0,06**	**homogeneous suppression** ⬇ **T=0,1**
**82**	**P62937**	PPIA_HUMAN	Peptidyl-prolyl cis-trans isomerase A	4, 11, 12, 14, 17, **19, 20, 21**	Cyclophilin A is involved in protein folding, assembly, transportation [84-89]	**homogeneous suppression** ⬇ **3x T=0,06**	Individual reaction ⬆ ➜ ⬇
**28**			not identified protein			**highly upregulated in several MKs T=0,06**	Individual reaction ⬆ ⬇
**53**			not identified protein			**highly upregulated in several MKs T=0,06**	Individual reaction ⬆ ⬇
**142**	**P08670**	VIME_HUMAN	Vimentin	1, 2, 11, 14, 18, **19, 20, 21**	Microfilamental network cell-migration related protein [60,76,90-97]	⬇ **2x T=0,09**	Individual reaction ⬆ ➜ ⬇
**62, 85 93-95**	**P00915**	CAH1_HUMAN	Carbonic anhydrase I	5, 11, 18 **19, 20, 21**	Energy metabolism related protein [98-104]	⬇ **2x T=0,10**	Individual reaction ⬆ ⬇
**143**	**P28838**	AMPL_HUMAN	Cytosol aminopeptidase	4, 11, 14 **19, 20, 21**	Regulatory protein-modification enzyme [105-109]	**individual induction **⬆ **T=0,1**	**homogeneous suppression** ⬇ **T=0,1**
**135**	**P49411**	EFTU_HUMAN	Elongation factor Tu, mitochondrial precursor	7, **20**	Mitochondrial protein synthesis machinery, critical role to maintain the translational fidelity [110,111]	**homogeneous suppression** ⬇ **4x T=0,1**	Individual reaction ⬆ ➜ ⬇
**45-46**	**P52566**	GDIS_HUMAN	Rho GDP-dissociation inhibitor 2 (Rho GDIß)	1, 2, 11, 12, 14, 17, **19, 20, 21**	LyDGI plays a role in the onset of apoptosis and cell migration [11,112-116]	**homogeneous upregulation **⬆ **T=0,1**	Individual reaction ⬆ ⬇
**148**	**P63104**	1433Z_HUMAN	14-3-3 protein zeta/delta (protein kinase C inhibitor)	11, 12, 14, 17, 18, **19, 20, 21**	Cell-cycle checkpoint, stress response protein [117-119]	**homogeneous suppression** ⬇ **2,5x T=0,1**	**homogeneous suppression** ⬇ **T=0,001**
**67**	**P06702**	S10A9_HUMAN	Protein S100-A9, Calgranulin	2, 11, 14, 18, **19, 20, 21**	Ca^2+^-dependent cell-migration related protein [11,120-127]	⬆ **2,5x T=0,11**	Individual reaction ⬆ ➜ ⬇
**110**			not identified protein			**highly upregulated in several MKsT=0,11**	Individual reaction ⬆ ⬇
**123**	**P07237**	PDIA1_HUMAN	Protein disulfide-isomerase precursor, PDI	4, 14, 9, 17, 18, **20, 21**	Stress-related protein modification enzyme [60,128-131]	⬇ **2,5x T=0,12**	Individual reaction ⬆ ⬇
**104**			not identified protein			**highly upregulated in several MKsT=0,12**	Individual reaction ⬆ ⬇
**CATEGORY B: non-significantly altered expression profiles in patients *versus *controls**							
**131**	**P78371**	TCPB_HUMAN	T-complex protein 1 subunit beta	4, **20, 21**	A member of chaperons family [132,133]	individual upregulation ⬆ 2x T=0,15	**homogeneous suppression** ⬇ **T=0,05**
**19-21, 39**	**P60709**	ACTB_HUMAN	Actin, cytoplasmic 1 (Beta-actin)	1, 2, 11, 14, 18, **19, 20, 21**	Microfilamental network protein [11]	slightly increased ⬆ 1,5x T=0,16	Individual reaction ⬆ ➜ ⬇
**97**	**P60174**	TPIS_HUMAN	Triosephosphate isomerase	5, 7, **19, 20, 21**	Energy metabolism related protein [134-137]	individual upregulation ⬆ 2x T=0,2	Individual reaction ⬆ ⬇
**44**		ANXA1-HUMAN	Annexin A1 (Calpactin II)	9, 11, 14, 17, 18, **19, 20, 21**	Ca^2+^-dependent phospholipid-binding proteins, potential anti-inflammatory activity [138-141]	individual upregulation ⬆ 2x T=0,2	**homogeneous induction **⬆ **T=0,1**
**80**	**P05109**	S10A8_HUMAN	Protein S100-A8, Calgranulin	2, 11, 14, 18, **19, 20, 21**	Ca^2+^-dependent cell-migration / tumour related protein [11,120-127]	homogeneous ⬆ 2,0x T=0,24	Individual suppression ⬇
**37**	**P47756**	CAPZB_HUMAN	F-actin-capping protein subunit beta (CapZ beta)	1, 2, 11, 14, 18, **19, 20, 21**	Microfilamental network protein [142-145]	slightly increased ⬆ T=0,2	Individual reaction ⬆ ⬇
**137**	**P30041**	PRDX6_HUMAN	Peroxiredoxin-6	9, 10, 11, 14, 17, 18, **19, 20, 21**	Multifuctional anti-oxidant, defence, tumour-invasion and metastases related protein [146-150]	slightly increased T=0,2	Individual reaction ⬆ ⬇
**9-11**	**P02679**	FIBG_HUMAN	Fibrinogen gamma chain	11, 17, **19, 20, 21**	Microfilamental network cell-migration related protein [151-157]	homogeneous ⬆ 1,5x T=0,24	Individual reaction ⬆ ➜ ⬇
**130**	**P10809**	CH60_HUMAN	60 kDa heat shock protein	4, 5, 7, 11, 13, 17, **19, 20, 21**	Mitochondrial stress response protein,protein-folding [158-166]	slightly increased homogeneous level T=0,25	**homogeneous suppression** ⬇ **T=0,1**
**36**	**Q5U077**	Q5U077_HUMAN	L-lactate dehydrogenase B	5, 7, 11, **19, 20, 21**	Energy metabolism related protein [134,167-172]	slightly increased ⬆ 1,5x	Individual reaction ⬆ ⬇
**122**	**Q96C61**	Q96C61_HUMAN	FLNA protein	1, 2, 11, 14, **19, 20, 21**	Filamin A - actin binding protein has essential role in intercellular junctions [173-178]	homogeneous ⬆ 1,5x	Individual reaction ⬆ ⬇
**151**	**P07996**	TSP1_HUMAN	Thrombospondin-1 precursor	2, 15, 11, 14, 17, **19, 20, 21**	The matricellular protein regulating cell adhesion and motility during tissue remodelling, in fibrogenesis & angiogenesis [179-189]	Individual induction ⬆ T=0,29	Individual reaction ⬆ ⬇
**CATEGORY C: individual group-heterogeneous expression profiles in patients *versus *homogeneous one in controls**							
**86,87**	**P00918**	CAH2_HUMAN	Carbonic anhydrase II	5, 11, 18, **19, 20, 21**	Energy metabolism related protein [98-100,103,104,190,191]	Individual heterogeneous	Individual reaction ⬆ ⬇
**103**	**P02675**	FIBB_HUMAN	Fibrinogen beta chain precursor	11, 17, **19, 20, 21**	Microfilamental network cell-migration related protein [151-157]	Individual heterogeneous	Individual reaction ⬆ ➜ ⬇
**117-120**	**O75083**	WDR1_HUMAN	WD repeat-containing protein 1	4, 12, 11, 14, **20**	Cell-cycle and proteolytic machinery related protein [189,192]	Individual heterogeneous	Individual reaction ⬆ ➜ ⬇
**126**	**P28331**	NUAM_HUMAN	NADH-ubiquinone oxidoreductase 75 kDa	5, 7, 9, 11, 14, **19, 20, 21**	Mitochondrial energy metabolism related protein [193-197]	highly heterogeneous	Individual reaction ⬆ ⬇
**127**	**P08133**	ANXA6_HUMAN	Annexin A6 (P70)	2, 8, 11, 14, 16, 17, **19, 20, 21**	Membrane architecture and signalling protein [127,198-201]	Individual induction	Individual induction **⬆ **
**128**	**P11142**	HSP7C_HUMAN	Heat shock cognate 71 kDa protein	4, 5, 11, 13, 14, 17, 18, **19, 20, 21**	Stress response protein,chaperone, ATPase [202-206]	Individual heterogeneous	Individual reaction ⬆ ➜ ⬇
**144**	**Q53XX6**	ATPA_HUMAN	ATP-synthase, H+ transporting mitochondrial protein	5, 7, 8, 11, 18, **19, 20, 21**	Mitochondrial energy metabolism related protein [207-212]	Individual heterogeneous	Individual reaction ⬆ ⬇
**147**	**P31946**	1433B_HUMAN	14-3-3 protein beta/alpha (protein- kinase-C inhibitor)	4, 11, 12, 14, 17, **19, 20, 21**	Cell-cycle checkpoint, stress response protein [118,213-216]	highly heterogeneous	**homogeneous suppression **⬇ **T=0,001**
**152**	**P14780**	MMP9_HUMAN	Matrix metalloproteinase-9	11, 14, 15, 18, **19, 20, 21**	MMP9 Multifunctional tissue-remodeling protein [217-222]	highly heterogeneous	Individual reaction ⬆ ⬇
**CATEGORY D: similar expression-profiles among patients and controls**							
**1-5**	**P18206**	VINC_HUMAN		Vinculin	Cytoskeletal assembly associated protein	similar	Individual reaction ⬆ ⬇
**6-8, 17, 34, 38, 63, 105, 109, 111**				not identified protein spots		similar	Individual reaction ⬆ ⬇
**12,13, 32, 33, 43, 47, 48, 98**	**P60709**	ACTB_HUMAN		Actin, cytoplasmic 1 (Beta-actin)	Microfilamental network protein	similar	Individual reaction ⬆ ⬇
**14-15**	**P68363**	TBA1B_HUMAN		Tubulin alpha- chain	Microtubule network protein	similar	Individual reaction ⬆ ⬇
**16**	**P06576**	ATPB_HUMAN		ATP synthase subunit beta, mitochondrial precursor	Mitochondrial energy metabolism related protein	similar	Individual reaction ⬆ ⬇
**18**	**P07437**	TBB2_HUMAN		Tubulin beta-2 chain	Microfilamental network protein	similar	Individual reaction ⬆ ➜ ⬇
**29-31, 51, 52, 154–61, 64, 79, 81, 83, 84, 89-92**	**P13645**	K1C10_HUMAN		Keratin, type I cytoskeletal 10	Microfilamental network protein	similar	Individual reaction ⬆ ⬇
**40, 41**	**P63261**	ACTG_HUMAN		Actin, cytoplasmic 2 (Gamma-actin)	Microfilamental network protein	similar	Individual reaction ⬆ ⬇
**42**		Q6FHP5_HUMAN		PHB protein	Prohibitin - negative regulator of cell proliferation and may be a tumor suppressor. Mutations in PHB have been linked to sporadic breast cancer.	similar	homogeneous suppression ⬇
**49-50**	**P67936**	TPM4_HUMAN		Tropomyosin alpha-4 chain	Microfilamental network protein	similar	Individual reaction ⬆ ⬇
**88**	**P02768**	ALBU_HUMAN		Serum albumin	Extracellular transport/carrier protein	similar	Individual reaction ⬆ ⬇
**96**	**P18669**	PGAM1_HUMAN		Phosphoglycerate mutase 1	Energy metabolism related protein	similar	**homogeneous suppression **⬇ **T=0,1**
**99**	**P00558**	PGK1_HUMAN		Phosphoglycerate kinase 1	Energy metabolism related protein	similar	Individual reaction ⬆ ➜ ⬇
**100**	**P68871**	HBB_HUMAN		Hemoglobin subunit beta	Oxygen carrier	similar	Individual reaction ⬆ ⬇
**101, 102, 106**	**P06733**	ENOA_HUMAN		Alpha-enolase	multifunctional glycolytic enzyme	similar	Individual reaction ⬆ ➜ ⬇
**107**	**P14618**	KPYM_HUMAN		Pyruvate kinase, isozymes M1/M2	Energy metabolism related protein	similar	Individual reaction ⬆ ➜ ⬇
**125**	**P14625**	ENPL_HUMAN		Endoplasmin precursor (94-kDa glucose-regulated protein)	Signal transduction pathways associated with endoplasmic reticulum stress	similar	**homogeneous suppression** ⬇ **T=0,1**
**129**	**P13796**	PLSL_HUMAN		Plastin-2	Microfilamental network protein	similar	**homogeneous suppression **⬇ **T=0,02**
**132**	**Q53QM2**	Q53QM2_HUMAN		Hypothetical protein ACTR3	Currently uncharacterized protein	similar	**homogeneous suppression **⬇ **T=0,1**
**133**	**Q6IAT1**	Q6IAT1_HUMAN		GDI2 protein (GDP dissociation inhibitor 2)	Regulatory protein in the functional cycle and recycling of Rab GTPases	similar	Individual suppression ⬇
**134**		UQCR1_HUMAN		Reductase complex core protein I	Ubiquinol-cytochrome C- reductase, mitochondrial processing peptidase Beta-family	similar	Individual reaction ⬆ ⬇
**138**	**P09211**	GSTP1_HUMAN		Glutathione S-transferase P (GST class-pi)	Stress response and anti-oxidant defence protein	similar	**homogeneous induction **⬆ **T=0,07**
**139**	**P07741**	APT_HUMAN		Adenine phosphoribosyl-transferase	Nucleotide metabolism	similar	Individual reaction ⬆ ➜ ⬇
**140**	**P11021**	GRP78_HUMAN		78 kDa glucose-regulated protein precursor (GRP 78)	Energy metabolism related protein	similar	Individual reaction ⬆ ➜ ⬇
**145**	**P13716**	HEM2_HUMAN		Delta-aminolevulinic acid dehydratase	anti-oxidant defence and detoxification pathways	similar	**homogeneous suppression **⬇ **T=0,07**
**146**	**Q5JQI4**	HSP71_HUMAN		Heat shock 70 kDa protein 1A	Stress response protein	similar	Individual reaction ⬆ ⬇
**149**	**Q06323**	PSME1_HUMAN		Proteasome activator complex subunit 1	The activator binds to proteasome 20S & enhances peptidase activity, e.g. under stress conditions	similar	Individual reaction ⬆ ⬇
**150**	**P00491**	PNPH_HUMAN		Purine nucleoside phosphorylase	Nucleotide- and nucleoside turnover, detoxification pathway	similar	Individual suppression ⬇
**153**	**P12429**	ANXA3_HUMAN		Annexin A3	Membrane architecture and signalling protein	similar	Individual reaction ⬆ ➜ ⬇
**154**		VDAC1_HUMAN		Voltage-dependent anion-selective channel protein 1	Membrane protein, regulation of cell growth / death via redox-control	similar	Individual induction ⬆
**158**		Q9Y3F5_HUMAN		A6-related hypothetical protein	Twinfilin-2, Protein tyrosine kinase 9-like, actin-binding protein involved in motile and morphological processes	similar	**Homogeneous suppression **⬇ **T=0,1**

**Annotation to Table**1**:** 158 spots have been distinguished by protein mapping as stably expressed (i.e. by all members of the group) in circulating leucocytes of the group with breast cancer patients. Altogether 74 proteins have been identified within 158 spots. The protein mapping image is demonstrated in Figure 5. The spot number in the map (**Spot number**) and corresponding accession number (**Access number**) and name (**Accession name**) received from the *SwissProt* database is provided in the table together with the name of the identified protein (**Protein name**) in accordance with the current protein nomenclature. The column “**Classification**” provides information about the function(s) currently known for each protein. The corresponding number of the functional group(s) is/are provided in the column “**Functional group number”**; the designation of the functional group with the corresponding number can be found in the separate Table 2. The regulation manner (up / down regulation) and the severity of the expression profile alterations under the cancer condition have been qualified and quantified *versus* the values in the control group; the resulting information is provided in the column “**Profile alterations *versus *controls”**. In accordance to the expression profile alterations, every mapped protein has been assigned to one of the altogether four CATEGORIES built-up as follows: A = 22 proteins with the statistically significant alterations in the expression profiles under the cancer condition compared to the control group (T≤0,1); B = 12 with the statistically non-significant alterations in the expression profiles under the cancer condition compared to the control group; C = 9 proteins with the expression profiles altered individually with highly heterogeneous expression profiles within the patient group *versus* stable expression levels within the control group; D = 31 proteins with similar expression-profiles within both patient and control groups of comparison. Further, under the cancer condition, the expression alterations as the reaction towards the applied radiotherapy has been qualified (up / down regulation) and quantified as it is summarised for each protein in the column “**Alterations under radiotherapy**”. The resulting statistics is provided here: 14 proteins homogeneously (group-significantly) suppressed (**⬇ **), 4 proteins homogeneously (group-significantly) induced (⬆ ), 4 proteins individually (group-non-significantly) suppressed (**⬇ **), 2 proteins individually (group-non-significantly) induced (⬆ ), 33 individually up- or down-regulated proteins (⬆ ⬇ ), and 17 proteins with individual up-/or down-/or unchanged regulation (⬆ ➜ ⬇ ) have been profiled under radiotherapy.

Concomitantly to the protein identification, the functional classification has been performed. The list of functional groups is provided with the separate Table 2.

**Table 2:** Systematic overview of the integrative panel of proteins/functional groups involved in the breast cancer specific molecular patterns in blood cells

**Nr.**	**Functional group**	**Relevance for breast cancer in tissue [reference]**	**Relevance for breast cancer in blood [reference]**
**1**	microfilamental network-associated and cytoskeletal-assembly proteins	[48,223,224]	[11]
**2**	cell motility, migration & adhesion	[225-227]	[11]
**3**	nucleoside / nucleotide turnover & metabolism	[228,229]	
**4**	protein metabolism (regulatory protein-synthesis & protein-modification enzymes, chaperons)	[230,231]	[231]
**5**	energy metabolism	[232-236]	[232,236]
**6**	vitamin metabolism	[237,238]	
**7**	mitochondrial proteins	[239-241]	[239,241]
**8**	channels, membrane-architecture and intercellular-junction proteins	[242]	
**9**	anti-oxidant defence / red-ox control	[243-246]	[245]
**10**	detoxification proteins	[247]	
**11**	stress-response / -protection related protein	[75,248-250]	
**12**	cell-cycle machinery proteins	[251-253]	
**13**	heat-shock proteins	[254-258]	
**14**	apoptosis-related proteins / protection against apoptosis	[259-261]	[262,263]
**15**	tissue-remodelling enzymes	[21,264-268]	
**16**	extra-cellular transport & carrier-proteins	[258,269,270]	
**17**	signal-transduction proteins / signalling pathways	[271-274]	
**18**	longevity / ageing related proteins	[275-278]	
**19**	**inflammation related / anti-inflammatory proteins**	[14,21,279]	
**20**	**(breast) cancer related inhibitor / promoter**	see references to individual proteins listed in the **Table**1	
**21**	**cancer invasion and regulator of metastases formation**	see references to individual proteins listed in **Table**1,1	

#### Breast cancer specific expression patterns as potential candidates for the predictive-diagnostic biomarker panel

The expression profiles under the cancer condition have been quantified *versus* the control group with benign and no breast tumours detected [11]. The resulting information is provided in Table 1. In accordance to statistical analysis, altogether four categories have been built-up as follows: A. statistically significant alterations in the expression profiles under the cancer condition compared to the control group; B. statistically non-significant alterations in the expression profiles under the cancer condition compared to the control group; C. expression levels altered individually with highly heterogeneous expression profiles within the patient group *versus* stable expression levels within the control group; D similar expression-profiles within both patient and control groups of comparison. Here detected pathology specific patterns might be further considered for the creation of the biomarker panel of high predictive power in diagnosing of the breast cancer development.

#### Group-specific versus individual therapy response: potential prognostic tool by proteomic blood tests?

As it is summarised in Table 1, the reaction towards the standardised radiotherapy has been quantified at the level of the protein expression rates in circulating leucocytes. The resulting statistical analysis demonstrated following patterns: 14 proteins were significantly suppressed and 4 proteins were significantly induced in all patients tested. In contrast, further 4 proteins were individually (group-non-significantly) suppressed and 2 proteins individually (group-non-significantly) induced. However, for the absolute majority (50) of the proteins measured strictly individual post-therapeutic regulation (up- / down or unchanged) was monitored. These findings motivates a creation of the “follow-up” projects to learn more about “molecular signature” of the patient beneficial therapy response as the potential prognostic tool.

### What do we learn by the function of proteins involved in the breast cancer specific expression alterations in blood?

Below listed groups (see Table 2) have been created according to the function(s) of individual proteins identified through the breast cancer specific profiles in circulating leucocytes (see Table 1). The literature sources relevant for the issue are listed in the Table 1 respectively to the functional groups. What do we learn from the exercise?

➢ According to the content summarised in the Table 2, it is evident that the breast cancer specific protein profiles affect a spectrum of the central biological activities in and of the cell.

➢ The multifactorial impacts of the disease are evident.

➢ Certainly there are effective interactions among individual functional groups: several proteins are involved and play a (key) role at least in two but frequently in a much higher number of the functional groups listed.

➢ All the proteins with expression rates altered under the breast cancer condition as described in this article, have been reported to stay in a kind of relation to cancer / breast cancer / metastatic activity. Moreover, some of the combinations of the proteins presented here have been already reported in relation to breast/cancer.

➢ However, the particular value of this article is in the systematic overview of the integrative panel of proteins/functional groups involved in the breast cancer specific molecular patterns in blood cells.

➢ Furthermore, the tool is obviously of high importance in favour of non-invasive prediction of breast cancer, since only very few literature sources could be found for breast cancer blood biomarker/patterns.

### Personalised treatments of the manifested breast cancer: where are we now?

During the last years several biomarkers as well as molecular factors have made their way into clinical routine. Extensive translational research, new mathematical models and computer-based analysis resulted in validated markers that allow personalised decision making for each individual patient already nowadays. Below we summarise the actualities and factors that have recently been shown to provide additional prognostic or predictive information and can finally spare ineffective or even harmful treatments (e.g. chemotherapy) and promote approaches tailored to the patient.

Clinicopathological factors, such as the histological subtype, tumour grade as well as the expression of the receptors for oestrogen, progesterone and HER2 belong to the most established evidence for making decisions over individualised therapeutic approaches. Therefrom, the expression levels of oestrogen receptor and HER2 are currently the best known predictive and prognostic biomarkers for individualised breast cancer therapy [280]. Increased expression rates of HER2 is the valid biomarker for an unfavourable prognosis in breast cancer management [281,282]. Furthermore, retrospective studies revealed a functional link between the level of HER2 expression and an individual patient response towards endocrine therapy and sensitivity to *taxanes* and *anthracyclines*[283-285]. However, the highest impact of HER2 in the clinical practice is its predictive and prognostic value indicating a response to *trastuzumab* and *pertuzumab* as well as to *lapatinib* (an inhibitor of the tyrosine kinase domain within HER1 and HER2 sequences) [286-288].

Further, a potential clinical utilisation of novel biomarkers dealing with the enzymatic complexes of cell proliferation, such as ki67 and uPA/PAI-1, is on the horizon. Hence, an elevated expression of ki67 is a potent marker for aggressive tumour types and a consequently poor prognosis [289,290]. Several studies demonstrated an association of ki67 expressional level with the quality of patient response towards chemotherapy and endocrine therapy [291,292]. Consequently, ki67 has been included into the *St. Gallen Consensus Recommendations* to stratify breast tumours according to the level of proliferation [293]. In primary breast cancer, independent prognostic factors uPA/PAI-1 indicates a level of the tumour invasion and metastatic disease that is of particular value for treatments of the node-negative breast cancer [294,295]. Both factors have reached highest level of evidence (LOI-1) and have been recommended for the classification of the groups of risk in making decisions for treatments of the node-negative breast cancer [296,297].

The central role in creating an individual risk profile receives the computer assistance. For example, *Adjuvant!Online* is an internet-based algorithm aiming at prediction of the recurrence free survival and total survival over 10 years [298]. This programme takes into consideration the best established clinical and pathology-specific contributing risk factors such as tumour size, nodal involvement, histology, hormone receptor status and age in combination with co-morbidities registered. *Adjuvant!Online* may be potentially utilised to prognose individual risks and benefits of endocrine therapy and / or variants of chemotherapy regimes proposed individually for the patients [299-301]. An alternative programme is *PREDICT+* for the efficacy prediction based on individual HER2 parameters and hormone status [302-304].

Gene expression profiles receive more and more recognition in the overall breast cancer management including typification, prediction, prognosis and therapy regiments. Based on the common gene expression patterns, the molecular breast cancer subtypes have been grouped into five classes, namely Luminal-A, Luminal-B, Basal-like, ErbB2-like and normal-like ones [305,306]. Therefrom, each intrinsic breast cancer subtype is characterised by an individual prognostic relevance, patterns of the metastatic disease and typical response to single therapy approaches [307-309]. Consequently, these intrinsic subtypes have been included into the *St. Gallen Consensus Therapy Recommendations*[293]. For the first time in the history of breast cancer management, the *Consensus Expert Panel* decides on the individualisation of the adjuvant therapy considering the molecular patterns as follows:

➢ sole endocrine therapy in Luminal-A-cancers

➢ endocrine therapy in combination with chemotherapy in Luminal-B cancers

➢ sole chemotherapy in Basal-Like subtypes, and

➢ chemotherapy in combination with anti-HER2-treatment in ErbB2-like breast cancer.

Further, there are commercially available multi-gene assays that may be used to prognose individual recurrence scores and may assist in making decisions on single treatment regiments. The most common are *MammaPrint* and *Oncotype DX* assays [310,311]. Therewith, *MammaPrint* is able to distinguish breast cancer patients with a good prognosis to avoid unnecessary and even harmful treatments [312,313]. In contrast, the identified cohort of patients with a poor prognosis are more likely to achieve beneficial results by neo-adjuvant chemotherapy [314]. *Oncotype DX* is developed for patients with hormone receptor positive tumours undergoing endocrine treatment with *tamoxifen*. Therefore, this test identifies patients with a low risk of the tumour recurrence, who would not benefit from additionally applied adjuvant chemotherapy [315]. An add-value of the Oncotype DX application as evident for the node-positive disease, since patients with high tumour-recurrence scores may well benefit from anthracycline-based chemotherapy [316]. Both assays are currently under the prospective study in the *MINDACT trial* (*MammaPrint*) and *TAILORx trial* (*Oncotype DX*) to validate their overall clinical utility for the personalised application of adjuvant chemotherapeutic approaches [317,318].

## Recommendations and outlook

Diagnosis and treatments of breast cancer metastasis disease (BCMD) are extremely challenging that prompts a development of emerging technologies for the effective prevention of breast cancer. Therefore, the overall task is formulated as the integrative medical approach of the multimodal diagnostics, disease specific biomarker-patterns, individual patient profiles, creation of medical records and treatments tailored to the person. In this context, a minimally invasive breast cancer risk assessment appears to be a plausible approach for early / predictive diagnosis of cancer pre-stages and targeted treatments before the clinical onset of BCMD.

The multimodal diagnostiscs represents a model-based examination procedure with several levels of examination resulting in the extended patient profiles and medical records which should obligatory include an interview with the patient / a questionnaire form filled in for pathology relevant information, medical imaging, laboratory diagnostics and evaluation of pathology relevant risk factors. For the laboratory diagnostics it is highly recommended to use valid blood tests for the detection of the stage specific molecular patterns in activated leucocytes as explained above.

For the application of adjuvant therapeutic approaches, our ethical responsibility requests a carefully created balance between risks and benefits to justify the individually made decisions. A predictive genetic testing should be fixed by law to determine effective treatment options by evaluating efficacy, e.g. in the case of cytochrome P450 *CYP2D6* genotyping to decide on *tamoxifen* application tailored to the patient.

Innovative medical records should be, further, developed to cover current deficits in the above listed clinical and laboratory expertise and to create individual patient profiles utilising mathematical modelling and integrative bioinformatics.

## Competing interests

The authors declare that they have no competing interests.

## Authors’ contributions

OG created the concept of the project, made the data interpretation and drafted the article. KY carried out the molecular biological studies. VC participated in the creation of the concept of the article. DT supervised the patients recruitment and data collection at the Department of Radiology. MB supervised the patients recruitment and data collection at the Department of Obstetrics and Gynaecology. MD contributed to the drafting of the paper. WK supervised the project at the Department of Obstetrics and Gynaecology. HS supervised the project at the Department of Radiology. All authors read and approved the final manuscript.

## References

[B1] WHO Cancer[http://www.who.int/mediacentre/factsheets/fs297/en/]

[B2] Jemal A Siegel R Ward E Hao Y Xu J Thun MJ Cancer Statistics, 2009 CA Cancer J Clin 2009 59 225 249 10.3322/caac.20006 19474385

[B3] National Cancer Institute at the National Institutes of Health Breast Cancerhttp://www.cancer.gov/cancertopics/types/breast

[B4] Lu J Steeg PS Price JE Krishnamurthy S Mani SA Reuben J Cristofanilli M Dontu G Bidaut L Valero V Hortobagyi GN Yu D Breast cancer metastasis: challenges and opportunities Cancer Res 2009 69 4951 4953 10.1158/0008-5472.CAN-09-0099 19470768

[B5] Ross JS Hortobagyi GN Molecular Oncology of Breast Cancer 2004 Ma: Jones & Bartlett Pub

[B6] Hayashi N Yamauchi H Role of circulating tumor cells and disseminated tumor cells in primary breast cancer Breast Cancer 2012 19 110 117 10.1007/s12282-011-0282-5 21643809

[B7] Allard WJ Matera J Miller MC Repollet M Connelly MC Rao C Tibbe AGJ Uhr JW Terstappen LWMM Tumor cells circulate in the peripheral blood of all major carcinomas but not in healthy subjects or patients with nonmalignant diseases Clin Cancer Res 2004 10 6897 6904 10.1158/1078-0432.CCR-04-0378 15501967

[B8] Alix-Panabières C Riethdorf S Pantel K Circulating tumor cells and bone marrow micrometastasis Clin Cancer Res 2008 14 5013 5021 10.1158/1078-0432.CCR-07-5125 18698019

[B9] Ignatiadis M Xenidis N Perraki M Apostolaki S Politaki E Kafousi M Stathopoulos EN Stathopoulou A Lianidou E Chlouverakis G Sotiriou C Georgoulias V Mavroudis D Different prognostic value of cytokeratin-19 mRNA positive circulating tumor cells according to estrogen receptor and HER2 status in early-stage breast cancer J Clin Oncol 2007 25 5194 5202 10.1200/JCO.2007.11.7762 17954712

[B10] Nguyen DX Massagué J Genetic determinants of cancer metastasis Nat Rev Genet 2007 8 341 352 17440531 10.1038/nrg2101

[B11] Braun M Fountoulakis M Yeghiazaryan K Schild HH Kuhn W Golubnitschaja O Golubnitschaja O How realistic are non-invasive approaches in breast cancer prediction? Predictive Diagnostics and Personalized Treatment: Dream or Reality 2009 New York: Nova Science Publishers Inc 433 446

[B12] Vorbach C Capecchi MR Penninger JM Evolution of the mammary gland from the innate immune system? Bioessays 2006 28 606 616 10.1002/bies.20423 16700061

[B13] Howard BA Gusterson BA Human breast development J Mammary Gland Biol Neoplasia 2000 5 119 137 10.1023/A:1026487120779 11149569

[B14] DeNardo DG Coussens LM Inflammation and breast cancer. Balancing immune response: crosstalk between adaptive and innate immune cells during breast cancer progression Breast Cancer Res 2007 9 212 10.1186/bcr1746 17705880 PMC2206719

[B15] Dunn GP Old LJ Schreiber RD The immunobiology of cancer immunosurveillance and immunoediting Immunity 2004 21 137 148 10.1016/j.immuni.2004.07.017 15308095

[B16] Coronella-Wood JA Hersh EM Naturally occurring B-cell responses to breast cancer Cancer Immunol Immunother 2003 52 715 738 10.1007/s00262-003-0409-4 12920480 PMC11033039

[B17] Wong PY Staren ED Tereshkova N Braun DP Functional analysis of tumor-infiltrating leukocytes in breast cancer patients J Surg Res 1998 76 95 103 10.1006/jsre.1998.5301 9695747

[B18] Coussens LM Werb Z Inflammation and cancer Nature 2002 420 860 867 10.1038/nature01322 12490959 PMC2803035

[B19] Balkwill F Charles KA Mantovani A Smoldering and polarized inflammation in the initiation and promotion of malignant disease Cancer Cell 2005 7 211 217 10.1016/j.ccr.2005.02.013 15766659

[B20] Wiseman BS Werb Z Stromal effects on mammary gland development and breast cancer Science 2002 296 1046 1049 10.1126/science.1067431 12004111 PMC2788989

[B21] Hojilla CV Wood GA Khokha R Inflammation and breast cancer: metalloproteinases as common effectors of inflammation and extracellular matrix breakdown in breast cancer Breast Cancer Res 2008 10 205 10.1186/bcr1980 18394187 PMC2397522

[B22] Golubnitschaja O Costigliola V Common origin but individual outcomes: time for new guidelines in personalized healthcare Personalized Med 2010 7 561 568 10.2217/pme.10.42 29776246

[B23] Ross JS Golubnitschaja O Integrated diagnostics and personalized therapeutics in oncology Predictive Diagnostics and Personalized Treatment: Dream or Reality 2009 New York: Nova Science Publishers Inc 399 431

[B24] Yeghiazaryan K Braun M Mamlouk S Schild HH Golubnitschaja O Are side-effects of irradiation predictable for treatment of breast cancer patients? Predictive Diagnostics and Personalized Treatment: Dream or Reality 2009 New York: Nova Science Publishers Inc 447 456

[B25] Gahan P Circulating nucleic acids in plasma and serum: diagnosis and prognosis in cancer EPMA J 2010 1 503 512 10.1007/s13167-010-0021-6 23199092 PMC3405334

[B26] Mallmann M Staratschek-Jox A Rudlowski C Braun M Gaarz A Wolfgarten M Kuhn W Schultze J Prediction and prognosis: impact of gene expression profiling in personalized treatment of breast cancer patients EPMA J 2010 1 421 437 10.1007/s13167-010-0044-z 23199086 PMC3405335

[B27] Debald M Wolfgarten M Walgenbach-Brünagel G Kuhn W Braun M Non-invasive proteomics—thinking about personalized breast cancer screening and treatment EPMA J 2010 1 413 420 10.1007/s13167-010-0039-9 23199085 PMC3405342

[B28] Yeghiazaryan K Mamlouk S Trog D Moenkemann H Braun M Kuhn W Schild H Golubnitschaja O Irradiated breast cancer patients demonstrate subgroup-specific regularities in protein expression patterns of circulating leukocytes Cancer Genomics Proteomics 2007 4 411 418 18204204

[B29] Golubnitschaja O Cell cycle checkpoints: the role and evaluation for early diagnosis of senescence, cardiovascular, cancer, and neurodegenerative diseases Amino Acids 2007 32 359 371 10.1007/s00726-006-0473-0 17136506

[B30] NIH / NCI The Breast Cancer Risk Assessment Tool[http://www.cancer.gov/bcrisktool/]

[B31] Cebioglu M Schild HH Golubnitschaja O Diabetes mellitus as a risk factor for cancer: stress or viral etiology? Infect Disord Drug Targets 2008 8 76 87 10.2174/187152608784746501 18537703

[B32] Golubnitschaja O Costigliola V Changing long-held beliefs is never easy: A Proposal for multimodal approaches in female healthcare - An Integrative view Healthcare Overview: New Perspectives 2012 Dordrecht Heidelberg New York London: Springer 251 268

[B33] NIH / NCI Sleep Disorders [http://www.cancer.gov/cancertopics/pdq/supportivecare/sleepdisorders/HealthProfessional/page1/AllPages]

[B34] Richter K Acker J Kamcev N Bajraktarov S Piehl A Niklewski G Recommendations for the prevention of breast cancer in shift workers EPMA J 2011 2 351 356 10.1007/s13167-011-0126-6 23199173 PMC3405398

[B35] Golubnitschaja O Moenkemann H Kim K Mozaffari MS DNA damage and expression of checkpoint genes p21(WAF1/CIP1) and 14-3-3 sigma in taurine-deficient cardiomyocytes Biochem Pharmacol 2003 66 511 517 10.1016/S0006-2952(03)00285-5 12907251

[B36] Yeghiazaryan K Cebioglu M Braun M Kuhn W Schild HH Golubnitschaja O Noninvasive subcellular imaging in breast cancer risk assessment: construction of diagnostic windows Personalized Med 2011 8 321 330 10.2217/pme.11.17 29783528

[B37] Bechtel W Bauer G Catalase Protects Tumor Cells from Apoptosis Induction by Intercellular ROS Signaling Anticancer Res 2009 29 4541 4557 20032403

[B38] Bai J Cederbaum AI Catalase Protects HepG2 Cells from Apoptosis Induced by DNA-damaging Agents by Accelerating the Degradation of p53 J Biol Chem 2003 278 4660 4667 10.1074/jbc.M206273200 12468545

[B39] Nishikawa M Tamada A Hyoudou K Umeyama Y Takahashi Y Kobayashi Y Kumai H Ishida E Staud F Yabe Y Takakura Y Yamashita F Hashida M Inhibition of experimental hepatic metastasis by targeted delivery of catalase in mice Clin Exp Metastasis 2004 21 213 221 15387371 10.1023/b:clin.0000037706.13747.5e

[B40] Jang B-C Paik J-H Kim S-P Shin D-H Song D-K Park J-G Suh M-H Park J-W Suh S-I Catalase induced expression of inflammatory mediators via activation of NF-kappaB, PI3K/AKT, p70S6K, and JNKs in BV2 microglia Cell Signal 2005 17 625 633 10.1016/j.cellsig.2004.10.001 15683737

[B41] Ahn J Gammon MD Santella RM Gaudet MM Britton JA Teitelbaum SL Terry MB Nowell S Davis W Garza C Neugut AI Ambrosone CB Associations between breast cancer risk and the catalase genotype, fruit and vegetable consumption, and supplement use Am J Epidemiol 2005 162 943 952 10.1093/aje/kwi306 16192345

[B42] Goh J Enns L Fatemie S Hopkins H Morton J Pettan-Brewer C Ladiges W Mitochondrial targeted catalase suppresses invasive breast cancer in mice BMC Cancer 2011 11 191 10.1186/1471-2407-11-191 21605372 PMC3123323

[B43] Zou L Jaramillo M Whaley D Wells A Panchapakesa V Das T Roy P Profilin-1 is a negative regulator of mammary carcinoma aggressiveness Br J Cancer 2007 97 1361 1371 10.1038/sj.bjc.6604038 17940506 PMC2360229

[B44] Masui O White NM Desouza LV Krakovska O Matta A Metias S Khalil B Romaschin AD Honey RJ Stewart R Pace K Bjarnason GA Siu KW Yousef GM Quantitative proteomic analysis in metastatic renal cell carcinoma reveals a unique set of proteins with potential prognostic significance Mol Cell Proteomics 2013 12 132 144 10.1074/mcp.M112.020701 23082029 PMC3536894

[B45] Roy P Jacobson K Overexpression of profilin reduces the migration of invasive breast cancer cells Cell Motil Cytoskeleton 2004 57 84 95 10.1002/cm.10160 14691948

[B46] Wittenmayer N Jandrig B Rothkegel M Schlüter K Arnold W Haensch W Scherneck S Jockusch BM Tumor suppressor activity of profilin requires a functional actin binding site Mol Biol Cell 2004 15 1600 1608 10.1091/mbc.E03-12-0873 14767055 PMC379259

[B47] Cao Y Motomura K Ohtsuru A Matsumoto T Yamashita S Kosaka M Profilin gene expression and regulation in a temperature-sensitive breast cancer cell line: tsFT101 Pflugers Arch 1997 434 341 345 10.1007/s004240050406 9211798

[B48] Janke J Schlüter K Jandrig B Theile M Kölble K Arnold W Grinstein E Schwartz A Estevéz-Schwarz L Schlag PM Jockusch BM Scherneck S Suppression of tumorigenicity in breast cancer cells by the microfilament protein profilin 1 J Exp Med 2000 191 1675 1686 10.1084/jem.191.10.1675 10811861 PMC2193149

[B49] Rabinovitz I Simpson K Cress AE, Nagle RB The actin cytoskeleton and metastasis Cell Adhesion and Cytoskeletal Molecules in Metastasis 2006 Dordrecht: Springer 69 90

[B50] Renz M Betz B Niederacher D Bender HG Langowski J Invasive breast cancer cells exhibit increased mobility of the actin-binding protein CapG Int J Cancer 2008 122 1476 1482 18059028 10.1002/ijc.23215

[B51] Kim JY Lee YG Kim M-Y Byeon SE Rhee MH Park J Katz DR Chain BM Cho JY Src-mediated regulation of inflammatory responses by actin polymerization Biochem Pharmacol 2010 79 431 443 10.1016/j.bcp.2009.09.016 19769947

[B52] Pellegrin S Mellor H Actin stress fibres J Cell Sci 2007 120 3491 3499 10.1242/jcs.018473 17928305

[B53] Sotiriou C Pusztai L Gene-expression signatures in breast cancer N Engl J Med 2009 360 790 800 10.1056/NEJMra0801289 19228622

[B54] Dai E Stewart M Ritchie B Mesaeli N Raha S Kolodziejczyk D Hobman ML Liu LY Etches W Nation N Michalak M Lucas A Calreticulin, a Potential Vascular Regulatory Protein, Reduces Intimal Hyperplasia After Arterial Injury Arterioscler Thromb Vasc Biol 1997 17 2359 2368 10.1161/01.ATV.17.11.2359 9409202

[B55] Watanabe K Ohira H Orikasa H Saito K Kanno K Shioya Y Obara K Sato Y Anti-calreticulin antibodies in patients with inflammatory bowel disease Fukushima J Med Sci 2006 52 1 11 16995349 10.5387/fms.52.1

[B56] Alur M Nguyen MM Eggener SE Jiang F Dadras SS Stern J Kimm S Roehl K Kozlowski J Pins M Michalak M Dhir R Wang Z Suppressive roles of calreticulin in prostate cancer growth and metastasis Am J Pathol 2009 175 882 890 10.2353/ajpath.2009.080417 19608864 PMC2716982

[B57] Kageyama S Isono T Iwaki H Wakabayashi Y Okada Y Kontani K Yoshimura K Terai A Arai Y Yoshiki T Identification by proteomic analysis of calreticulin as a marker for bladder cancer and evaluation of the diagnostic accuracy of its detection in urine Clin Chem 2004 50 857 866 10.1373/clinchem.2003.027425 14764641

[B58] Lwin Z-M Guo C Salim A Yip GW-C Chew F-T Nan J Thike AA Tan P-H Bay B-H Clinicopathological significance of calreticulin in breast invasive ductal carcinoma Mod Pathol 2010 23 1559 1566 10.1038/modpathol.2010.173 20834237

[B59] Liu H Bowes RC van de Water B Sillence C Nagelkerke JF Stevens JL Endoplasmic Reticulum Chaperones GRP78 and Calreticulin Prevent Oxidative Stress, Ca2+ Disturbances, and Cell Death in Renal Epithelial Cells J Biol Chem 1997 272 21751 21759 10.1074/jbc.272.35.21751 9268304

[B60] Chahed K Kabbage M Ehret-Sabatier L Lemaitre-Guillier C Remadi S Hoebeke J Chouchane L Expression of fibrinogen E-fragment and fibrin E-fragment is inhibited in the human infiltrating ductal carcinoma of the breast: the two-dimensional electrophoresis and MALDI-TOF-mass spectrometry analyses Int J Oncol 2005 27 1425 1431 16211239

[B61] Quandt KS Hultquist DE Flavin reductase: sequence of cDNA from bovine liver and tissue distribution Proc Natl Acad Sci U S A 1994 91 9322 9326 10.1073/pnas.91.20.9322 7937764 PMC44804

[B62] Lu H Chen J Planko L Zigrino P Klein-Hitpass L Magin TM Induction of inflammatory cytokines by a keratin mutation and their repression by a small molecule in a mouse model for EBS J Invest Dermatol 2007 127 2781 2789 17581617 10.1038/sj.jid.5700918

[B63] Lyda MH Tetef M Carter NH Ikle D Weiss LM Arber DA Keratin immunohistochemistry detects clinically significant metastases in bone marrow biopsy specimens in women with lobular breast carcinoma Am J Surg Pathol 2000 24 1593 1599 10.1097/00000478-200012000-00002 11117779

[B64] Hendrix MJC Seftor EA Chu Y-W Trevor KT Seftor REB Role of intermediate filaments in migration, invasion and metastasis Cancer Metastasis Rev 1996 15 507 525 10.1007/BF00054016 9034607

[B65] Paccione RJ Miyazaki H Patel V Waseem A Gutkind JS Zehner ZE Yeudall WA Keratin down-regulation in vimentin-positive cancer cells is reversible by vimentin RNA interference, which inhibits growth and motility Mol Cancer Ther 2008 7 2894 2903 10.1158/1535-7163.MCT-08-0450 18790770

[B66] Russell D Andrews PD James J Lane EB Mechanical stress induces profound remodelling of keratin filaments and cell junctions in epidermolysis bullosa simplex keratinocytes J Cell Sci 2004 117 5233 5243 10.1242/jcs.01407 15454576

[B67] Sivaramakrishnan S Schneider JL Sitikov A Goldman RD Ridge KM Shear Stress Induced Reorganization of the Keratin Intermediate Filament Network Requires Phosphorylation by Protein Kinase C ζ Mol Biol Cell 2009 20 2755 2765 10.1091/mbc.E08-10-1028 19357195 PMC2688554

[B68] Tung JJ Kitajewski J Chloride intracellular channel 1 functions in endothelial cell growth and migration J Angiogenes Res 2010 2 23 10.1186/2040-2384-2-23 21040583 PMC2993651

[B69] Wang J-W Peng S-Y Li J-T Wang Y Zhang Z-P Cheng Y Cheng D-Q Weng W-H Wu X-S Fei X-Z Quan Z-W Li J-Y Li S-G Liu Y-B Identification of metastasis-associated proteins involved in gallbladder carcinoma metastasis by proteomic analysis and functional exploration of chloride intracellular channel 1 Cancer Lett 2009 281 71 81 10.1016/j.canlet.2009.02.020 19299076

[B70] Suginta W Karoulias N Aitken A Ashley RH Chloride intracellular channel protein CLIC4 (p64H1) binds directly to brain dynamin I in a complex containing actin, tubulin and 14-3-3 isoforms Biochem J 2001 359 55 64 10.1042/0264-6021:3590055 11563969 PMC1222121

[B71] Suh KS Mutoh M Nagashima K Fernandez-Salas E Edwards LE Hayes DD Crutchley JM Marin KG Dumont RA Levy JM Cheng C Garfield S Yuspa SH The organellular chloride channel protein CLIC4/mtCLIC translocates to the nucleus in response to cellular stress and accelerates apoptosis J Biol Chem 2004 279 4632 4641 14610078 10.1074/jbc.M311632200

[B72] Beliakoff J Whitesell L Hsp90: an emerging target for breast cancer therapy Anticancer Drugs 2004 15 651 662 10.1097/01.cad.0000136876.11928.be 15269596

[B73] Yaobin Tong W Zhu Y Study on HSP70, 90 mRNA gene expression in peripheral blood mononuclear cells with steroid-resistant asthmatics Zhonghua Jie He He Hu Xi Za Zhi 1998 21 289 292 11326954

[B74] Njemini R Bautmans I Onyema O Puyvelde KV Demanet C Mets T Circulating Heat Shock Protein 70 in Health, Aging and Disease BMC Immunol 2011 12 24 10.1186/1471-2172-12-24 21443787 PMC3074541

[B75] Ciocca DR Calderwood SK Heat shock proteins in cancer: diagnostic, prognostic, predictive, and treatment implications Cell Stress Chaperones 2005 10 86 103 10.1379/CSC-99r.1 16038406 PMC1176476

[B76] Zhang M-H Lee J-S Kim H-J Jin D-I Kim J-I Lee K-J Seo J-S HSP90 protects apoptotic cleavage of vimentin in geldanamycin-induced apoptosis Mol Cell Biochem 2006 281 111 121 10.1007/s11010-006-0638-x 16328963

[B77] Liu J Chen J Yu L Tian Y Cui X Yan Q Fu L Inhibitory effect of ginsenoside-Rg3 on lung metastasis of mouse melanoma transfected with ribonuclease inhibitor Zhonghua Zhong Liu Za Zhi 2004 26 722 725 15733388

[B78] Chen J Ou-Yang X Gao J Zhu J He X Rong J Knockdown of ribonuclease inhibitor expression with siRNA in non-invasive bladder cancer cell line BIU-87 promotes growth and metastasis potentials Mol Cell Biochem 2010 349 83 95 21125316 10.1007/s11010-010-0663-7

[B79] Dickson KA Effect of the Ribonuclease Inhibitor on the Biological Activity of Pancreatic-Type Ribonucleases. PhD thesis 2006 Madison: University of Wisconsin

[B80] Moenner M Vosoghi M Ryazantsev S Glitz DG Ribonuclease inhibitor protein of human erythrocytes: characterization, loss of activity in response to oxidative stress, and association with Heinz bodies Blood Cells Mol Dis 1998 24 149 164 10.1006/bcmd.1998.0182 9628852

[B81] Fominaya JM Hofsteenge J Inactivation of ribonuclease inhibitor by thiol-disulfide exchange J Biol Chem 1992 267 24655 24660 1447207

[B82] Chen J-X Gao Y Liu J-W Tian Y-X Zhao J Cui X-Y Antitumor effects of human ribonuclease inhibitor gene transfected on B16 melanoma cells Int J Biochem Cell Biol 2005 37 1219 1231 10.1016/j.biocel.2004.11.020 15778086

[B83] Tumor Research Center The Influences of Human Placental Ribonuclease Inhibitor Mutants on Their Biological Activities[http://www.tumorres.com/brain-tumor/10397.htm]

[B84] Wulf G Garg P Liou Y-C Iglehart D Lu KP Modeling breast cancer in vivo and ex vivo reveals an essential role of Pin1 in tumorigenesis EMBO J 2004 23 3397 3407 10.1038/sj.emboj.7600323 15257284 PMC514501

[B85] Bao L Kimzey A Sauter G Sowadski JM Lu KP Wang D-G Prevalent overexpression of prolyl isomerase Pin1 in human cancers Am J Pathol 2004 164 1727 1737 10.1016/S0002-9440(10)63731-5 15111319 PMC1615639

[B86] Song F Zhang X Ren X-B Zhu P Xu J Wang L Li Y-F Zhong N Ru Q Zhang D-W Jiang J-L Xia B Chen Z-N Cyclophilin A (CyPA) induces chemotaxis independent of its peptidylprolyl cis-trans isomerase activity: direct binding between CyPA and the ectodomain of CD147 J Biol Chem 2011 286 8197 8203 10.1074/jbc.C110.181347 21245143 PMC3048706

[B87] Dourlen P Ando K Hamdane M Begard S Buée L Galas MC The peptidyl prolyl cis/trans isomerase Pin1 downregulates the Inhibitor of Apoptosis Protein Survivin Biochim Biophys Acta 2007 1773 1428 1437 10.1016/j.bbamcr.2007.05.012 17624454

[B88] Yue F Wang L-S Xia L Wang X-L Feng B Lu A-G Chen G-Q Zheng M-H Modulated T-complex protein 1 ζ and peptidyl-prolyl cis-trans isomerase B are two novel indicators for evaluating lymph node metastasis in colorectal cancer: Evidence from proteomics and bioinformatics Proteomics Clin Appl 2009 3 1225 1235 10.1002/prca.200900028 21136946

[B89] Wulf GM Ryo A Wulf GG Lee SW Niu T Petkova V Lu KP Pin1 is overexpressed in breast cancer and cooperates with Ras signaling in increasing the transcriptional activity of c-Jun towards cyclin D1 EMBO J 2001 20 3459 3472 10.1093/emboj/20.13.3459 11432833 PMC125530

[B90] Thaiparambil J Bender L Kline E Ganesh T Snyder J Liotta D Marcus A Vimentin: A Novel Chemopreventive Target for Breast Cancer Metastasis Cancer Res 2010 69 5063 5063

[B91] Kokkinos MI Wafai R Wong MK Newgreen DF Thompson EW Waltham M Vimentin and epithelial-mesenchymal transition in human breast cancer–observations in vitro and in vivo Cells Tissues Organs (Print) 2007 185 191 203 10.1159/000101320 17587825

[B92] Kusinska RU Kordek R Pluciennik E Bednarek AK Piekarski JH Potemski P Does vimentin help to delineate the so-called “basal type breast cancer”? J Exp Clin Cancer Res 2009 28 118 10.1186/1756-9966-28-118 19695088 PMC2738661

[B93] Vuoriluoto K Haugen H Kiviluoto S Mpindi J-P Nevo J Gjerdrum C Tiron C Lorens JB Ivaska J Vimentin regulates EMT induction by Slug and oncogenic H-Ras and migration by governing Axl expression in breast cancer Oncogene 2011 30 1436 1448 10.1038/onc.2010.509 21057535

[B94] Korsching E Packeisen J Liedtke C Hungermann D Wülfing P van Diest PJ Brandt B Boecker W Buerger H The origin of vimentin expression in invasive breast cancer: epithelial-mesenchymal transition, myoepithelial histogenesis or histogenesis from progenitor cells with bilinear differentiation potential? J Pathol 2005 206 451 457 10.1002/path.1797 15906273

[B95] Moisan E Chiasson S Girard D The intriguing normal acute inflammatory response in mice lacking vimentin Clin Exp Immunol 2007 150 158 168 10.1111/j.1365-2249.2007.03460.x 17680824 PMC2219279

[B96] Mor-Vaknin N Punturieri A Sitwala K Markovitz DM Vimentin is secreted by activated macrophages Nat Cell Biol 2002 5 59 63 12483219 10.1038/ncb898

[B97] Wei J Xu G Wu M Zhang Y Li Q Liu P Zhu T Song A Zhao L Han Z Chen G Wang S Meng L Zhou J Lu Y Wang S Ma D Overexpression of vimentin contributes to prostate cancer invasion and metastasis via src regulation Anticancer Res 2008 28 327 334 18383865

[B98] Tafreshi NK Bui MM Bishop K Lloyd MC Enkemann SA Lopez AS Abrahams D Carter BW Vagner J Grobmyer SR Gobmyer SR Gillies RJ Morse DL Noninvasive detection of breast cancer lymph node metastasis using carbonic anhydrases IX and XII targeted imaging probes Clin Cancer Res 2012 18 207 219 10.1158/1078-0432.CCR-11-0238 22016510 PMC4130557

[B99] Pastorekova S Zatovicova M Pastorek J Cancer-associated carbonic anhydrases and their inhibition Curr Pharm Des 2008 14 685 698 10.2174/138161208783877893 18336315

[B100] Bekku S Mochizuki H Yamamoto T Ueno H Takayama E Tadakuma T Expression of carbonic anhydrase I or II and correlation to clinical aspects of colorectal cancer Hepatogastroenterology 2000 47 998 1001 11020863

[B101] Knudsen JF Carlsson U Hammarström P Sokol GH Cantilena LR The Cyclooxygenase-2 Inhibitor Celecoxib Is a Potent Inhibitor of Human Carbonic Anhydrase II Inflammation 2004 28 285 290 10.1007/s10753-004-6052-1 16134002

[B102] Radhakrishnan R Sluka KA Acetazolamide, a carbonic anhydrase inhibitor, reverses inflammation-induced thermal hyperalgesia in rats J Pharmacol Exp Ther 2005 313 921 927 15743922 10.1124/jpet.104.082776

[B103] Yasukawa Z Sato C Kitajima K Identification of an inflammation-inducible serum protein recognized by anti-disialic acid antibodies as carbonic anhydrase II J Biochem 2007 141 429 441 17298961 10.1093/jb/mvm047

[B104] Bodh S Kumar V Raina U Ghosh B Thakar M Inflammatory glaucoma Oman J Ophthalmol 2011 4 3 10.4103/0974-620X.77655 21713239 PMC3110445

[B105] Martínez JM Prieto I Ramírez MJ Cueva C Alba F Ramírez M Aminopeptidase Activities in Breast Cancer Tissue Clin Chem 1999 45 1797 1802 10508127

[B106] Sekine K Fujii H Abe F Nishikawa K Augmentation of death ligand-induced apoptosis by aminopeptidase inhibitors in human solid tumor cell lines Int J Cancer 2001 94 485 491 10.1002/ijc.1492 11745433

[B107] Varona A Blanco L López JI Gil J Agirregoitia E Irazusta J Larrinaga G Altered levels of acid, basic, and neutral peptidase activity and expression in human clear cell renal cell carcinoma Am J Physiol Renal Physiol 2007 292 F780 F788 16985214 10.1152/ajprenal.00148.2006

[B108] Bukowska A Tadje J Arndt M Wolke C Kähne T Bartsch J Faust J Neubert K Hashimoto Y Lendeckel U Transcriptional regulation of cytosol and membrane alanyl-aminopeptidase in human T cell subsets Biol Chem 2003 384 657 665 12751795 10.1515/BC.2003.073

[B109] Röhnert P Schmidt W Emmerlich P Goihl A Wrenger S Bank U Nordhoff K Täger M Ansorge S Reinhold D Striggow F Dipeptidyl peptidase IV, aminopeptidase N and DPIV/APN-like proteases in cerebral ischemia J Neuroinflammation 2012 9 44 10.1186/1742-2094-9-44 22373413 PMC3359160

[B110] Xu C Wang J Li J Fang R Expression of Elongation Factor (EF)-Tu Is Correlated with Prognosis of Gastric Adenocarcinomas Int J Mol Sci 2011 12 6645 6655 10.3390/ijms12106645 22072909 PMC3211000

[B111] Zheng G Peng F Ding R Yu Y Ouyang Y Chen Z Xiao Z He Z Identification of proteins responsible for the multiple drug resistance in 5-fluorouracil-induced breast cancer cell using proteomics analysis J Cancer Res Clin Oncol 2010 136 1477 1488 10.1007/s00432-010-0805-z 20700687 PMC11828137

[B112] Fritz G Just I Kaina B Rho GTPases are over-expressed in human tumors Int J Cancer 1999 81 682 687 10.1002/(SICI)1097-0215(19990531)81:5<682::AID-IJC2>3.0.CO;2-B 10328216

[B113] Seraj MJ Harding MA Gildea JJ Welch DR Theodorescu D The relationship of BRMS1 and RhoGDI2 gene expression to metastatic potential in lineage related human bladder cancer cell lines Clin Exp Metastasis 2000 18 519 525 10.1023/A:1011819621859 11592309

[B114] Fujita A Shida A Fujioka S Kurihara H Okamoto T Yanaga K Clinical significance of Rho GDP dissociation inhibitor 2 in colorectal carcinoma Int J Clin Oncol 2012 17 137 142 10.1007/s10147-011-0270-y 21698524

[B115] Moissoglu K McRoberts KS Meier JA Theodorescu D Schwartz MA Rho GDP dissociation inhibitor 2 suppresses metastasis via unconventional regulation of RhoGTPases Cancer Res 2009 69 2838 2844 10.1158/0008-5472.CAN-08-1397 19276387 PMC2701105

[B116] Zhang B Zhang Y Dagher M-C Shacter E Rho GDP Dissociation Inhibitor Protects Cancer Cells against Drug-Induced Apoptosis Cancer Res 2005 65 6054 6062 10.1158/0008-5472.CAN-05-0175 16024605

[B117] Vercoutter-Edouart AS Lemoine J Le Bourhis X Louis H Boilly B Nurcombe V Révillion F Peyrat JP Hondermarck H Proteomic analysis reveals that 14-3-3sigma is down-regulated in human breast cancer cells Cancer Res 2001 61 76 80 11196201

[B118] Pan Y Zhong L Zhou H Wang X Chen K Yang H Xiaokaiti Y Maimaiti A Jiang L Li X Roles of vimentin and 14-3-3 zeta/delta in the inhibitory effects of heparin on PC-3M cell proliferation and B16-F10-luc-G5 cells metastasis Acta Pharmacol Sin 2012 33 798 808 10.1038/aps.2012.42 22669117 PMC4010382

[B119] Wong TT Zhou L Li J Tong L Zhao SZ Li XR Yu SJ Koh SK Beuerman RW Proteomic profiling of inflammatory signaling molecules in the tears of patients on chronic glaucoma medication Invest Ophthalmol Vis Sci 2011 52 7385 7391 10.1167/iovs.10-6532 21697136

[B120] Croce K Gao H Wang Y Mooroka T Sakuma M Shi C Sukhova GK Packard RRS Hogg N Libby P Simon DI Myeloid-related protein-8/14 is critical for the biological response to vascular injury Circulation 2009 120 427 436 10.1161/CIRCULATIONAHA.108.814582 19620505 PMC3070397

[B121] Leach ST Mitchell HM Geczy CL Sherman PM Day AS S100 calgranulin proteins S100A8, S100A9 and S100A12 are expressed in the inflamed gastric mucosa of Helicobacter pylori-infected children Can J Gastroenterol 2008 22 461 464 18478131 10.1155/2008/308942 PMC2660800

[B122] Carlsson H Petersson S Enerbäck C Cluster analysis of S100 gene expression and genes correlating to psoriasin (S100A7) expression at different stages of breast cancer development Int J Oncol 2005 27 1473 1481 16273201

[B123] Kennedy RD Gorski JJ Quinn JE Stewart GE James CR Moore S Mulligan K Emberley ED Lioe TF Morrison PJ Mullan PB Reid G Johnston PG Watson PH Harkin DP BRCA1 and c-Myc associate to transcriptionally repress psoriasin, a DNA damage-inducible gene Cancer Res 2005 65 10265 10272 10.1158/0008-5472.CAN-05-1841 16288014

[B124] Rust R Visser L van der Leij J Harms G Blokzijl T Deloulme JC van der Vlies P Kamps W Kok K Lim M Poppema S van den Berg A High expression of calcium-binding proteins, S100A10, S100A11 and CALM2 in anaplastic large cell lymphoma Br J Haematol 2005 131 596 608 10.1111/j.1365-2141.2005.05816.x 16351635

[B125] Cross SS Hamdy FC Deloulme JC Rehman I Expression of S100 proteins in normal human tissues and common cancers using tissue microarrays: S100A6, S100A8, S100A9 and S100A11 are all overexpressed in common cancers Histopathology 2005 46 256 269 10.1111/j.1365-2559.2005.02097.x 15720411

[B126] Hermani A Hess J Servi BD Medunjanin S Grobholz R Trojan L Angel P Mayer D Calcium-Binding Proteins S100A8 and S100A9 as Novel Diagnostic Markers in Human Prostate Cancer Clin Cancer Res 2005 11 5146 5152 10.1158/1078-0432.CCR-05-0352 16033829

[B127] Bode G Lüken A Kerkhoff C Roth J Ludwig S Nacken W Interaction between S100A8/A9 and annexin A6 is involved in the calcium-induced cell surface exposition of S100A8/A9 J Biol Chem 2008 283 31776 31784 10.1074/jbc.M803908200 18786929

[B128] Lee H-H Lim C-A Cheong Y-T Singh M Gam L-H Comparison of protein expression profiles of different stages of lymph nodes metastasis in breast cancer Int J Biol Sci 2012 8 353 362 22393307 10.7150/ijbs.3157 PMC3291852

[B129] Rao KVK Boukli NM Samikkannu T Cubano LA Dakshayani BK Nair MP Proteomics Profiling and Cytotoxic Effect of Curcuma longa on Prostate Cancer Open Proteomics J 2011 4 1 11 10.2174/1875039701104010001

[B130] Goplen D Wang J Enger PØ Tysnes BB Terzis AJA Laerum OD Bjerkvig R Protein Disulfide Isomerase Expression Is Related to the Invasive Properties of Malignant Glioma Cancer Res 2006 66 9895 9902 10.1158/0008-5472.CAN-05-4589 17047051

[B131] Hoffstrom BG Kaplan A Letso R Schmid RS Turmel GJ Lo DC Stockwell BR Inhibitors of protein disulfide isomerase suppress apoptosis induced by misfolded proteins Nat Chem Biol 2010 6 900 906 10.1038/nchembio.467 21079601 PMC3018711

[B132] Satish L Johnson S Wang JH-C Post JC Ehrlich GD Kathju S Chaperonin Containing T-Complex Polypeptide Subunit Eta (CCT-eta) Is a Specific Regulator of Fibroblast Motility and Contractility PLoS One 2010 5 e10063 10.1371/journal.pone.0010063 20442790 PMC2862014

[B133] Wong STC Zhao H Molecular Diagnostic Methods for Predicting Brain Metastasis of Breast Cancer International Patent US 2012/0184560 A1

[B134] Poulsen N Andersen V Møller J Møller H Jessen F Purup S Larsen L Comparative analysis of inflamed and non-inflamed colon biopsies reveals strong proteomic inflammation profile in patients with ulcerative colitis BMC Gastroenterol 2012 12 76 10.1186/1471-230X-12-76 22726388 PMC3441502

[B135] Tamesa MS Kuramitsu Y Fujimoto M Maeda N Nagashima Y Tanaka T Yamamoto S Oka M Nakamura K Detection of autoantibodies against cyclophilin A and triosephosphate isomerase in sera from breast cancer patients by proteomic analysis Electrophoresis 2009 30 2168 2181 10.1002/elps.200800675 19582718

[B136] Dang Y Wang Z Guo Y Yang J Xing Z Mu L Zhang X Ding Z Overexpression of triosephosphate isomerase inhibits proliferation of chicken embryonal fibroblast cells Asian Pac J Cancer Prev 2011 12 3479 3482 22471501

[B137] Zhang X Xiao Z Li C Xiao Z Yang F Li D Li M Li F Chen Z Triosephosphate isomerase and peroxiredoxin 6, two novel serum markers for human lung squamous cell carcinoma Cancer Sci 2009 100 2396 2401 10.1111/j.1349-7006.2009.01314.x 19737146 PMC11159988

[B138] Ang EZ-F Nguyen HT Sim H-L Putti TC Lim LHK Annexin-1 Regulates Growth Arrest Induced by High Levels of Estrogen in MCF-7 Breast Cancer Cells Mol Cancer Res 2009 7 266 274 10.1158/1541-7786.MCR-08-0147 19208747

[B139] Nair S Hande MP Lim LHK Annexin-1 protects MCF7 breast cancer cells against heat-induced growth arrest and DNA damage Cancer Lett 2010 294 111 117 10.1016/j.canlet.2010.01.026 20163912

[B140] Zhang Z Huang L Zhao W Rigas B Annexin 1 induced by anti-inflammatory drugs binds to NF-κB inhibiting its activation: Anticancer effects in vitro and in vivo Cancer Res 2010 70 2379 2388 10.1158/0008-5472.CAN-09-4204 20215502 PMC2953961

[B141] Peers SH Smillie F Elderfield AJ Flower RJ Glucocorticoid-and non-glucocorticoid induction of lipocortins (annexins) 1 and 2 in rat peritoneal leucocytes in vivo Br J Pharmacol 1993 108 66 72 10.1111/j.1476-5381.1993.tb13441.x 8428216 PMC1907693

[B142] Mojtahedi Z Erfani N Ghaderi A Comparative Proteomics Analysis of SKBR3 and MCF7 Breast Cancer Cell Lines Using Two Dimensional Electrophoresis: Ready to Build Postgenomics Capacity for OMICS R&D in Developing Countries? Curr Pharmacogenomics Personalized Med 2012 10 132 137 10.2174/187569212800626412

[B143] Májek P Reicheltová Z Stikarová J Suttnar J Sobotková A Dyr JE Proteome changes in platelets activated by arachidonic acid, collagen, and thrombin Proteome Sci 2010 8 56 21073729 10.1186/1477-5956-8-56 PMC2996359

[B144] España L Martín B Aragüés R Chiva C Oliva B Andreu D Sierra A Bcl-x(L)-mediated changes in metabolic pathways of breast cancer cells: from survival in the blood stream to organ-specific metastasis Am J Pathol 2005 167 1125 1137 10.1016/S0002-9440(10)61201-1 16192647 PMC1603674

[B145] Wang C-Y Chen J-K Wu Y-T Tsai M-J Shyue S-K Yang C-S Tzeng S-F Reduction in antioxidant enzyme expression and sustained inflammation enhance tissue damage in the subacute phase of spinal cord contusive injury J Biomed Sci 2011 18 13 10.1186/1423-0127-18-13 21299884 PMC3040708

[B146] Karihtala P Mäntyniemi A Kang SW Kinnula VL Soini Y Peroxiredoxins in Breast Carcinoma Clin Cancer Res 2003 9 3418 3424 12960131

[B147] Chang X-Z Li D-Q Hou Y-F Wu J Lu J-S Di G-H Jin W Ou Z-L Shen Z-Z Shao Z-M Identification of the functional role of peroxiredoxin 6 in the progression of breast cancer Breast Cancer Res 2007 9 R76 10.1186/bcr1789 17980029 PMC2246172

[B148] Ho J-N Lee SB Lee S-S Yoon SH Kang GY Hwang S-G Um H-D Phospholipase A2 Activity of Peroxiredoxin 6 Promotes Invasion and Metastasis of Lung Cancer Cells Mol Cancer Ther 2010 9 825 832 10.1158/1535-7163.MCT-09-0904 20354123

[B149] Wang Y Phelan SA Manevich Y Feinstein SI Fisher AB Transgenic Mice Overexpressing Peroxiredoxin 6 Show Increased Resistance to Lung Injury in Hyperoxia Am J Respir Cell Mol Biol 2006 34 481 486 10.1165/rcmb.2005-0333OC 16399955 PMC2644209

[B150] Sundar IK Chung S Hwang J-W Arunachalam G Cook S Yao H Mazur W Kinnula VL Fisher AB Rahman I Peroxiredoxin 6 differentially regulates acute and chronic cigarette smoke–mediated lung inflammatory response and injury Exp Lung Res 2010 36 451 462 10.3109/01902141003754128 20939758 PMC3062974

[B151] Somiari RI Somiari S Russell S Shriver CD Proteomics of breast carcinoma J Chromatogr B Analyt Technol Biomed Life Sci 2005 815 215 225 10.1016/j.jchromb.2004.11.012 15652811

[B152] Lee J Namkoong H Kim H Kim S Hwang D Na H Ha S-A Kim J-R Kim J Fibrinogen gamma-A chain precursor in CSF: a candidate biomarker for Alzheimer’s disease BMC Neurol 2007 7 14 10.1186/1471-2377-7-14 17565664 PMC1896177

[B153] Akakura N Hoogland C Takada YK Saegusa J Ye X Liu F-T Cheung AT-W Takada Y The COOH-terminal globular domain of fibrinogen gamma chain suppresses angiogenesis and tumor growth Cancer Res 2006 66 9691 9697 10.1158/0008-5472.CAN-06-1686 17018627

[B154] Palumbo JS Kombrinck KW Drew AF Grimes TS Kiser JH Degen JL Bugge TH Fibrinogen is an important determinant of the metastatic potential of circulating tumor cells Blood 2000 96 3302 3309 11071621

[B155] Lu C Mishra A Zhu YJ Meltzer P Cheng S-Y Genomic profiling of genes contributing to metastasis in a mouse model of thyroid follicular carcinoma Am J Cancer Res 2011 1 1 13 21562609 PMC3090007

[B156] Zhu W-L Fan B-L Liu D-L Zhu W-X Abnormal Expression of Fibrinogen Gamma (FGG) and Plasma Level of Fibrinogen in Patients with Hepatocellular Carcinoma Anticancer Res 2009 29 2531 2534 19596924

[B157] Du J Zheng J-H Chen X-S Yang Q Zhang Y-H Zhou L Yao X High preoperative plasma fibrinogen is an independent predictor of distant metastasis and poor prognosis in renal cell carcinoma Int J Clin Oncol 2012 10.1007/s10147-012-0412-x 22610754

[B158] Domeika M Domeika K Paavonen J Mårdh PA Witkin SS Humoral immune response to conserved epitopes of Chlamydia trachomatis and human 60-kDa heat-shock protein in women with pelvic inflammatory disease J Infect Dis 1998 177 714 719 10.1086/514218 9498452

[B159] Lohse AW Dienes HP Herkel J Hermann E van Eden W Büschenfelde KHM Expression of the 60 kDa heat shock protein in normal and inflamed liver J Hepatol 1993 19 159 166 10.1016/S0168-8278(05)80189-8 7905491

[B160] Chen W Syldath U Bellmann K Burkart V Kolb H Human 60-kDa heat-shock protein: a danger signal to the innate immune system J Immunol 1999 162 3212 3219 10092772

[B161] Pockley AG Heat Shock Proteins, Inflammation, and Cardiovascular Disease Circulation 2002 105 1012 1017 10.1161/hc0802.103729 11864934

[B162] Grundtman C Kreutmayer SB Almanzar G Wick MC Wick G Heat Shock Protein 60 and Immune Inflammatory Responses in Atherosclerosis Arterioscler Thromb Vasc Biol 2011 31 960 968 10.1161/ATVBAHA.110.217877 21508342 PMC3212728

[B163] Kligman I Grifo JA Witkin SS Expression of the 60 kDa heat shock protein in peritoneal fluids from women with endometriosis: implications for endometriosis-associated infertility Hum Reprod 1996 11 2736 2738 10.1093/oxfordjournals.humrep.a019200 9021381

[B164] Hwang YJ Lee SP Kim SY Choi YH Kim MJ Lee CH Lee JY Kim DY Expression of Heat Shock Protein 60 kDa Is Upregulated in Cervical Cancer Yonsei Med J 2009 50 399 406 10.3349/ymj.2009.50.3.399 19568603 PMC2703764

[B165] Barazi HO Zhou L Templeton NS Krutzsch HC Roberts DD Identification of Heat Shock Protein 60 as a Molecular Mediator of α3β1 Integrin Activation Cancer Res 2002 62 1541 1548 11888933

[B166] Cappello F Bellafiore M Palma A David S Marcianò V Bartolotta T Sciumè C Modica G Farina F Zummo G Bucchieri F 60KDa chaperonin (HSP60) is over-expressed during colorectal carcinogenesis Eur J Histochem 2003 47 105 110 12777205 10.4081/814

[B167] Shrinivasan A Poongothai A Rao C Srinivasulu M Vishnupriya S Serum Lactate Dehydrogenase (LDH) Levels In Breast Cancer Indian J Hum Genet 1999 5 21

[B168] Olinga P Merema MT de Jager MH Derks F Melgert BN Moshage H Slooff MJ Meijer DK Poelstra K Groothuis GM Rat liver slices as a tool to study LPS-induced inflammatory response in the liver J Hepatol 2001 35 187 194 10.1016/S0168-8278(01)00103-9 11580140

[B169] Chen Y Zhang H Xu A Li N Liu J Liu C Lv D Wu S Huang L Yang S He D Xiao X Elevation of serum l-lactate dehydrogenase B correlated with the clinical stage of lung cancer Lung Cancer 2006 54 95 102 10.1016/j.lungcan.2006.06.014 16890323

[B170] Hussien R Brooks GA Mitochondrial and plasma membrane lactate transporter and lactate dehydrogenase isoform expression in breast cancer cell lines Physiol Genomics 2011 43 255 264 10.1152/physiolgenomics.00177.2010 21177384 PMC3068517

[B171] Singh TD Barbhuiya MA Gupta S Shrivastav BR Jalaj V Agarwal N Tiwari PK Quantitative Assessment of Expression of Lactate Dehydrogenase and its Isoforms 3 and 4 may Serve as Useful Indicators of Progression of Gallbladder Cancer: A Pilot Study Indian J Clin Biochem 2011 26 146 153 10.1007/s12291-011-0117-3 22468041 PMC3107415

[B172] Chen Y Zhang H Xu A Liu J Li N Wu S Huang L He D Xiao X Identification and clinical evaluation of lung cancer serum biomarker L-lactate dehydrogenase B Zhonghua Jie He He Hu Xi Za Zhi 2007 30 577 581 17988549

[B173] Gay O Gilquin B Pitaval A Baudier J Refilins: A link between perinuclear actin bundle dynamics and mechanosensing signaling BioArchitecture 2011 1 245 249 10.4161/bioa.18246 22754617 PMC3384578

[B174] Le TH Protein dynamics in responder and non-responder solid tumor xenografts during oncolytic viral therapy. PhD thesis 2008 Bayerische Julius-Maximilians-Universität zu Würzburg

[B175] Yu N The role of the P2Y2 nucleotide receptors in vascular inflammation. PhD thesis 2007 University of Missouri

[B176] Zhong Z Yeow W-S Zou C Wassell R Wang C Pestell RG Quong JN Quong AA Cyclin D1/Cyclin-Dependent Kinase 4 Interacts with Filamin A and Affects the Migration and Invasion Potential of Breast Cancer Cells Cancer Res 2010 70 2105 2114 10.1158/0008-5472.CAN-08-1108 20179208 PMC2917898

[B177] Xu Y Bismar TA Su J Xu B Kristiansen G Varga Z Teng L Ingber DE Mammoto A Kumar R Alaoui-Jamali MA Filamin A regulates focal adhesion disassembly and suppresses breast cancer cell migration and invasion J Exp Med 2010 207 2421 2437 10.1084/jem.20100433 20937704 PMC2964581

[B178] Stevenson RP Veltman D Machesky LM Actin-bundling proteins in cancer progression at a glance J Cell Sci 2012 125 1073 1079 10.1242/jcs.093799 22492983

[B179] Gasparini G Toi M Biganzoli E Dittadi R Fanelli M Morabito A Boracchi P Gion M Thrombospondin-1 and −2 in Node-Negative Breast Cancer: Correlation with Angiogenic Factors, p53, Cathepsin D, Hormone Receptors and Prognosis Oncology 2001 60 72 80 10.1159/000055300 11150912

[B180] Hyder SM Liang Y Wu J Estrogen regulation of thrombospondin-1 in human breast cancer cells Int J Cancer 2009 125 1045 1053 10.1002/ijc.24373 19391135 PMC2755594

[B181] Sargiannidou I Zhou J Tuszynski GP The Role of Thrombospondin-1 in Tumor Progression Exp Biol Med (Maywood) 2001 226 726 733 11520937 10.1177/153537020222600803

[B182] Yee KO Connolly CM Duquette M Kazerounian S Washington R Lawler J The effect of thrombospondin-1 on breast cancer metastasis Breast Cancer Res Treat 2009 114 85 96 10.1007/s10549-008-9992-6 18409060 PMC2631620

[B183] Lopez-Dee Z Pidcock K Gutierrez LS Thrombospondin-1: Multiple Paths to Inflammation Mediators Inflamm 2011 2011 1 10 10.1155/2011/296069 PMC3134184 21765615

[B184] Rice AJ Steward MA Quinn CM Thrombospondin 1 protein expression relates to good prognostic indices in ductal carcinoma in situ of the breast J Clin Pathol 2002 55 921 925 10.1136/jcp.55.12.921 12461058 PMC1769827

[B185] Alaoui-Jamali MA Song DJ Benlimame N Yen L Deng X Hernandez-Perez M Wang T Regulation of multiple tumor microenvironment markers by overexpression of single or paired combinations of ErbB receptors Cancer Res 2003 63 3764 3774 12839972

[B186] Wang-Rodriguez J Urquidi V Rivard A Goodison S Elevated osteopontin and thrombospondin expression identifies malignant human breast carcinoma but is not indicative of metastatic status Breast Cancer Res 2003 5 R136 R143 10.1186/bcr620 12927044 PMC314424

[B187] Fontana A Filleur S Guglielmi J Frappart L Bruno-Bossio G Boissier S Cabon F Clézardin P Human breast tumors override the antiangiogenic effect of stromal thrombospondin-1 in vivo Int J Cancer 2005 116 686 691 10.1002/ijc.20584 15838828

[B188] Manni A Washington S Mauger D Hackett DA Verderame MF Cellular mechanisms mediating the anti-invasive properties of the ornithine decarboxylase inhibitor alpha-difluoromethylornithine (DFMO) in human breast cancer cells Clin Exp Metastasis 2004 21 461 467 10.1007/s10585-004-2724-3 15672871

[B189] Suh EJ Kabir MH Kang U-B Lee JW Yu J Noh D-Y Lee C Comparative profiling of plasma proteome from breast cancer patients reveals thrombospondin-1 and BRWD3 as serological biomarkers Exp Mol Med 2012 44 36 44 10.3858/emm.2012.44.1.003 22024541 PMC3277896

[B190] Leppilampi M Koistinen P Savolainen E-R Hannuksela J Parkkila A-K Niemelä O Pastoreková S Pastorek J Waheed A Sly WS Parkkila S Rajaniemi H The expression of carbonic anhydrase II in hematological malignancies Clin Cancer Res 2002 8 2240 2245 12114426

[B191] Parkkila S Rajaniemi H Parkkila AK Kivela J Waheed A Pastorekova S Pastorek J Sly WS Carbonic anhydrase inhibitor suppresses invasion of renal cancer cells in vitro Proc Natl Acad Sci U S A 2000 97 2220 2224 10.1073/pnas.040554897 10688890 PMC15781

[B192] Spiegelman VS Tang W Chan AM Igarashi M Aaronson SA Sassoon DA Katoh M Slaga TJ Fuchs SY Induction of homologue of Slimb ubiquitin ligase receptor by mitogen signaling J Biol Chem 2002 277 36624 36630 10.1074/jbc.M204524200 12151397

[B193] Tran M Tam D Bardia A Bhasin M Rowe GC Kher A Zsengeller ZK Akhavan-Sharif MR Khankin EV Saintgeniez M David S Burstein D Karumanchi SA Stillman IE Arany Z Parikh SM PGC-1α promotes recovery after acute kidney injury during systemic inflammation in mice J Clin Invest 2011 121 4003 4014 10.1172/JCI58662 21881206 PMC3195479

[B194] Gao B-B Phipps JA Bursell D Clermont AC Feener EP Angiotensin AT1 receptor antagonism ameliorates murine retinal proteome changes induced by diabetes J Proteome Res 2009 8 5541 5549 10.1021/pr9006415 19845401 PMC2798584

[B195] Putignani L Raffa S Pescosolido R Rizza T Del Chierico F Leone L Aimati L Signore F Carrozzo R Callea F Torrisi MR Grammatico P Preliminary evidences on mitochondrial injury and impaired oxidative metabolism in breast cancer Mitochondrion 2012 12 363 369 10.1016/j.mito.2012.02.003 22366096

[B196] Suhane S Berel D Ramanujan VK Biomarker signatures of mitochondrial NDUFS3 in invasive breast carcinoma Biochem Biophys Res Commun 2011 412 590 595 10.1016/j.bbrc.2011.08.003 21867691 PMC3171595

[B197] Kulawiec M Owens KM Singh KK mtDNA G10398A variant in African-American women with breast cancer provides resistance to apoptosis and promotes metastasis in mice J Hum Genet 2009 54 647 654 10.1038/jhg.2009.89 19763141 PMC2909846

[B198] Gerke V Moss SE Annexins: From Structure to Function Physiol Rev 2002 82 331 371 11917092 10.1152/physrev.00030.2001

[B199] Stogbauer F Weigert J Neumeier M Wanninger J Sporrer D Weber M Schaffler A Enrich C Wood P Grewal T Aslanidis C Buechler C Annexin A6 is highly abundant in monocytes of obese and type 2 diabetic individuals and is downregulated by adiponectin in vitro Exp Mol Med 2009 41 501 507 10.3858/emm.2009.41.7.055 19322030 PMC2721147

[B200] Vilá De Muga S Timpson P Cubells L Evans R Hayes TE Rentero C Hegemann A Reverter M Leschner J Pol A Tebar F Daly RJ Enrich C Grewal T Annexin A6 inhibits Ras signalling in breast cancer cells Oncogene 2009 28 363 377 10.1038/onc.2008.386 18850003

[B201] Sakwe AM Koumangoye R Guillory B Ochieng J Annexin A6 contributes to the invasiveness of breast carcinoma cells by influencing the organization and localization of functional focal adhesions Exp Cell Res 2011 317 823 837 10.1016/j.yexcr.2010.12.008 21185831 PMC3049817

[B202] Conroy SE Latchman DS Do heat shock proteins have a role in breast cancer? Br J Cancer 1996 74 717 721 10.1038/bjc.1996.427 8795573 PMC2074714

[B203] Nirdé P Derocq D Maynadier M Chambon M Basile I Gary-Bobo M Garcia M Heat shock cognate 70 protein secretion as a new growth arrest signal for cancer cells Oncogene 2010 29 117 127 10.1038/onc.2009.311 19802014 PMC2864961

[B204] Yokota S Chiba S Furuyama H Fujii N Cerebrospinal fluids containing anti-HSP70 autoantibodies from multiple sclerosis patients augment HSP70-induced proinflammatory cytokine production in monocytic cells J Neuroimmunol 2010 218 129 133 10.1016/j.jneuroim.2009.10.009 19919883

[B205] Gan L Liu D-B Lu H-F Long G-X Mei Q Hu G-Y Qiu H Hu G-Q Decreased expression of the carboxyl terminus of heat shock cognate 70 interacting protein in human gastric cancer and its clinical significance Onco Rep 2012 28 1392 1398 10.3892/or.2012.1957 22895543

[B206] Mizukami S Kajiwara C Ishikawa H Katayama I Yui K Udono H Both CD4+ and CD8+ T cell epitopes fused to heat shock cognate protein 70 (hsc70) can function to eradicate tumors Cancer Sci 2008 99 1008 1015 10.1111/j.1349-7006.2008.00788.x 18341654 PMC11160078

[B207] Hu J-Y Li C-L Wang Y-W Altered proteomic pattern in platelets of rats with sepsis Blood Cells Mol Dis 2012 48 30 35 10.1016/j.bcmd.2011.09.010 22014900

[B208] Hüttemann M Helling S Sanderson TH Sinkler C Samavati L Mahapatra G Varughese A Lu G Liu J Ramzan R Vogt S Grossman LI Doan JW Marcus K Lee I Regulation of mitochondrial respiration and apoptosis through cell signaling: cytochrome c oxidase and cytochrome c in ischemia/reperfusion injury and inflammation Biochim Biophys Acta 2012 1817 598 609 10.1016/j.bbabio.2011.07.001 21771582 PMC3229836

[B209] Huang T-C Chang H-Y Hsu C-H Kuo W-H Chang K-J Juan H-F Targeting therapy for breast carcinoma by ATP synthase inhibitor aurovertin B J Proteome Res 2008 7 1433 1444 10.1021/pr700742h 18275135

[B210] Willers IM Isidoro A Ortega AD Fernández PL Cuezva JM Selective inhibition of beta-F1-ATPase mRNA translation in human tumours Biochem J 2010 426 319 326 10.1042/BJ20091570 20028336

[B211] Pan J Sun L-C Tao Y-F Zhou Z Du X-L Peng L Feng X Wang J Li Y-P Liu L Wu S-Y Zhang Y-L Hu S-Y Zhao W-L Zhu X-M Lou G-L Ni J ATP synthase ecto-α-subunit: a novel therapeutic target for breast cancer J Transl Med 2011 9 211 10.1186/1479-5876-9-211 22152132 PMC3254596

[B212] Chang HJ Lee MR Hong S-H Yoo BC Shin Y-K Jeong JY Lim S-B Choi HS Jeong S-Y Park J-G Identification of mitochondrial FoF1-ATP synthase involved in liver metastasis of colorectal cancer Cancer Sci 2007 98 1184 1191 10.1111/j.1349-7006.2007.00527.x 17559425 PMC11159599

[B213] Hermeking H The 14-3-3 cancer connection Nat Rev Cancer 2003 3 931 943 14737123 10.1038/nrc1230

[B214] Butt AQ Ahmed S Maratha A Miggin SM 14-3-3{epsilon} and 14-3-3σ inhibit TLR-mediated pro-inflammatory cytokine induction J Biol Chem 2012 10.1074/jbc.M112.367490 PMC3493911 22984265

[B215] Hodgkinson VC Agarwal V ELFadl D Fox JN McManus PL Mahapatra TK Kneeshaw PJ Drew PJ Lind MJ Cawkwell L Pilot and feasibility study: comparative proteomic analysis by 2-DE MALDI TOF/TOF MS reveals 14-3-3 proteins as putative biomarkers of response to neoadjuvant chemotherapy in ER-positive breast cancer J Proteomics 2012 75 2745 2752 10.1016/j.jprot.2012.03.049 22498883

[B216] Minamida S Iwamura M Kodera Y Kawashima Y Tabata K Matsumoto K Fujita T Satoh T Maeda T Baba S 14-3-3 protein beta/alpha as a urinary biomarker for renal cell carcinoma: proteomic analysis of cyst fluid Anal Bioanal Chem 2011 401 245 252 10.1007/s00216-011-5057-5 21553213

[B217] Kim J-M Noh E-M Kwon K-B Kim J-S You Y-O Hwang J-K Hwang B-M Kim B-S Lee S-H Lee SJ Jung SH Youn HJ Lee Y-R Curcumin suppresses the TPA-induced invasion through inhibition of PKCα-dependent MMP-expression in MCF-7 human breast cancer cells Phytomedicine 2012 19 1085 1092 10.1016/j.phymed.2012.07.002 22921746

[B218] Debelec-Butuner B Alapinar C Varisli L Erbaykent-Tepedelen B Hamid SM Gonen-Korkmaz C Korkmaz KS Inflammation-mediated abrogation of androgen signaling: An in vitro model of prostate cell inflammation Mol Carcinog 2012 10.1002/mc.21948 22911881

[B219] Zhao Y Kong X Li X Yan S Yuan C Hu W Yang Q Metadherin mediates lipopolysaccharide-induced migration and invasion of breast cancer cells PLoS One 2011 6 e29363 10.1371/journal.pone.0029363 22195048 PMC3241708

[B220] Fatunmbi M Shelton J Aronica SM MMP-9 increases HER2/neu expression and alters apoptosis levels in human mammary epithelial cells (HMEC) Breast Cancer Res Treat 2012 135 519 530 10.1007/s10549-012-2191-5 22878890

[B221] Chakraborty S Kaur S Guha S Batra SK The multifaceted roles of neutrophil gelatinase associated lipocalin (NGAL) in inflammation and cancer Biochim Biophys Acta 2012 1826 129 169 22513004 10.1016/j.bbcan.2012.03.008 PMC3362670

[B222] Pellikainen JM Ropponen KM Kataja VV Kellokoski JK Eskelinen MJ Kosma V-M Expression of matrix metalloproteinase (MMP)-2 and MMP-9 in breast cancer with a special reference to activator protein-2, HER2, and prognosis Clin Cancer Res 2004 10 7621 7628 10.1158/1078-0432.CCR-04-1061 15569994

[B223] Dos Remedios CG Chhabra D Kekic M Dedova IV Tsubakihara M Berry DA Nosworthy NJ Actin binding proteins: regulation of cytoskeletal microfilaments Physiol Rev 2003 83 433 473 12663865 10.1152/physrev.00026.2002

[B224] Adam L Vadlamudi R Mandal M Chernoff J Kumar R Regulation of microfilament reorganization and invasiveness of breast cancer cells by kinase dead p21-activated kinase-1 J Biol Chem 2000 275 12041 12050 10.1074/jbc.275.16.12041 10766836

[B225] McSherry EA Brennan K Hudson L Hill ADK Hopkins AM Breast cancer cell migration is regulated through junctional adhesion molecule-A-mediated activation of Rap1 GTPase Breast Cancer Res 2011 13 R31 10.1186/bcr2853 21429211 PMC3219194

[B226] Huttenlocher A Horwitz AR Integrins in cell migration Cold Spring Harb Perspect Biol 2011 3 a005074 10.1101/cshperspect.a005074 21885598 PMC3181029

[B227] Friedl P Hegerfeldt Y Tusch M Collective cell migration in morphogenesis and cancer Int J Dev Biol 2004 48 441 449 10.1387/ijdb.041821pf 15349818

[B228] Bullinger D Neubauer H Fehm T Laufer S Gleiter CH Kammerer B Metabolic signature of breast cancer cell line MCF-7: profiling of modified nucleosides via LC-IT MS coupling BMC Biochem 2007 8 25 10.1186/1471-2091-8-25 18047657 PMC2219991

[B229] Buxton ILO Yokdang N Matz RM Purinergic mechanisms in breast cancer support intravasation, extravasation and angiogenesis Cancer Lett 2010 291 131 141 10.1016/j.canlet.2009.09.021 19926395 PMC2849889

[B230] Karve TM Cheema AK Small changes huge impact: the role of protein posttranslational modifications in cellular homeostasis and disease J Amino Acids 2011 2011 207691 22312457 10.4061/2011/207691 PMC3268018

[B231] Jin H Zangar RC Protein modifications as potential biomarkers in breast cancer Biomark Insights 2009 4 191 200 20072669 10.4137/bmi.s2557 PMC2805424

[B232] Vazquez-Martin A Oliveras-Ferraros C Cufí S Martin-Castillo B Menendez JA Metformin and energy metabolism in breast cancer: from insulin physiology to tumour-initiating stem cells Curr Mol Med 2010 10 674 691 10.2174/156652410792630625 20712585

[B233] Chen EI Hewel J Krueger JS Tiraby C Weber MR Kralli A Becker K Yates JR III Felding-Habermann B Adaptation of energy metabolism in breast cancer brain metastases Cancer Res 2007 67 1472 1486 10.1158/0008-5472.CAN-06-3137 17308085

[B234] Budczies J Denkert C Müller BM Brockmöller SF Klauschen F Györffy B Dietel M Richter-Ehrenstein C Marten U Salek RM Griffin JL Hilvo M Orešič M Wohlgemuth G Fiehn O Remodeling of central metabolism in invasive breast cancer compared to normal breast tissue - a GC-TOFMS based metabolomics study BMC Genomics 2012 13 334 10.1186/1471-2164-13-334 22823888 PMC3430598

[B235] Drabovich AP Pavlou MP Dimitromanolakis A Diamandis EP Quantitative analysis of energy metabolic pathways in MCF-7 breast cancer cells by selected reaction monitoring assay Mol Cell Proteomics 2012 11 422 434 10.1074/mcp.M111.015214 22535206 PMC3412972

[B236] Seyfried TN Shelton LM Cancer as a metabolic disease Nutr Metab (Lond) 2010 7 7 10.1186/1743-7075-7-7 20181022 PMC2845135

[B237] Mira-Y-Lopez R Zheng WL Kuppumbatti YS Rexer B Jing Y Ong DE Retinol conversion to retinoic acid is impaired in breast cancer cell lines relative to normal cells J Cell Physiol 2000 185 302 309 10.1002/1097-4652(200011)185:2<302::AID-JCP15>3.0.CO;2-# 11025452

[B238] Welsh J Vitamin D metabolism in mammary gland and breast cancer Mol Cell Endocrinol 2011 347 55 60 10.1016/j.mce.2011.05.020 21669251

[B239] Verma M Kagan J Sidransky D Srivastava S Proteomic analysis of cancer-cell mitochondria Nat Rev Cancer 2003 3 789 795 10.1038/nrc1192 14570046

[B240] Solazzo M Fantappiè O D’Amico M Sassoli C Tani A Cipriani G Bogani C Formigli L Mazzanti R Mitochondrial expression and functional activity of breast cancer resistance protein in different multiple drug-resistant cell lines Cancer Res 2009 69 7235 7242 10.1158/0008-5472.CAN-08-4315 19706772

[B241] Chen Y-W Chou H-C Lyu P-C Yin H-S Huang F-L Chang W-SW Fan C-Y Tu I-F Lai T-C Lin S-T Lu Y-C Wu C-L Huang S-H Chan H-L Mitochondrial proteomics analysis of tumorigenic and metastatic breast cancer markers Funct Integr Genomics 2011 11 225 239 10.1007/s10142-011-0210-y 21246238

[B242] Talhouk R On cell-matrix interactions in mammary gland development and breast cancer Cold Spring Harb Perspect Biol 2012 4 a013540 10.1101/cshperspect.a013540 22855728 PMC3405858

[B243] Manda G Nechifor MT Neagu T-M Reactive Oxygen Species, Cancer and Anti-Cancer Therapies Curr Chem Biol 2009 3 342 366 10.2174/187231309787158271

[B244] Acharya A Das I Chandhok D Saha T Redox regulation in cancer: a double-edged sword with therapeutic potential Oxid Med Cell Longev 2010 3 23 34 10.4161/oxim.3.1.10095 20716925 PMC2835886

[B245] Poole LB Hall A Nelson KJ Overview of peroxiredoxins in oxidant defense and redox regulation Curr Protoc Toxicol 2011 7 Unit7.9 21818754 10.1002/0471140856.tx0709s49 PMC3156475

[B246] Sainz RM Lombo F Mayo JC Radical Decisions in Cancer: Redox Control of Cell Growth and Death Cancers 2012 4 442 474 10.3390/cancers4020442 24213319 PMC3712695

[B247] Zhang D Tai LK Wong LL Chiu L-L Sethi SK Koay ESC Proteomic study reveals that proteins involved in metabolic and detoxification pathways are highly expressed in HER-2/neu-positive breast cancer Mol Cell Proteomics 2005 4 1686 1696 10.1074/mcp.M400221-MCP200 16048908

[B248] Stresing V Baltziskueta E Rubio N Blanco J Arriba M Valls J Janier M Clézardin P Sanz-Pamplona R Nieva C Marro M Dmitri P Sierra A Peroxiredoxin 2 specifically regulates the oxidative and metabolic stress response of human metastatic breast cancer cells in lungs Oncogene 2013 32 724 735 10.1038/onc.2012.93 22430214

[B249] Feldman DE Chauhan V Koong AC The unfolded protein response: a novel component of the hypoxic stress response in tumors Mol Cancer Res 2005 3 597 605 10.1158/1541-7786.MCR-05-0221 16317085

[B250] Curtis CD Thorngren DL Nardulli AM Immunohistochemical analysis of oxidative stress and DNA repair proteins in normal mammary and breast cancer tissues BMC Cancer 2010 10 9 10.1186/1471-2407-10-9 20064251 PMC2830938

[B251] Dressing GE Lange CA Integrated actions of progesterone receptor and cell cycle machinery regulate breast cancer cell proliferation Steroids 2009 74 573 576 10.1016/j.steroids.2008.12.001 19118566 PMC4871707

[B252] Roberti A Macaluso M Giordano A Giordano A, Normanno N Alterations in Cell Cycle Regulatory Genes in Breast Cancer Breast Cancer in the Post-Genomic Era 2009 Totowa, NJ: Humana Press 55 77

[B253] Abraham RT Cell cycle checkpoint signaling through the ATM and ATR kinases Genes Dev 2001 15 2177 2196 10.1101/gad.914401 11544175

[B254] Calderwood SK Heat shock proteins in breast cancer progression–a suitable case for treatment? Int J Hyperthermia 2010 26 681 685 10.3109/02656736.2010.490254 20653417 PMC3123897

[B255] Nylandsted J Rohde M Brand K Bastholm L Elling F Jäättelä M Selective depletion of heat shock protein 70 (Hsp70) activates a tumor-specific death program that is independent of caspases and bypasses Bcl-2 Proc Natl Acad Sci U S A 2000 97 7871 7876 10.1073/pnas.97.14.7871 10884417 PMC16637

[B256] Rehman A Chahal MS Tang X Bruce JE Pommier Y Daoud SS Proteomic identification of heat shock protein 90 as a candidate target for p53 mutation reactivation by PRIMA-1 in breast cancer cells Breast Cancer Res 2005 7 R765 R774 10.1186/bcr1290 16168122 PMC1242148

[B257] Caldewood SK, Sherman MY, Ciocca DR Heat Shock Proteins in Cancer 2010 Dordrecht: Springer

[B258] Schmitt E Gehrmann M Brunet M Multhoff G Garrido C Intracellular and extracellular functions of heat shock proteins: repercussions in cancer therapy J Leukoc Biol 2007 81 15 27 16931602 10.1189/jlb.0306167

[B259] Wu J Shao Z-M Shen Z-Z Lu J-S Han Q-X Fontana JA Barsky SH Significance of Apoptosis and Apoptotic-Related Proteins, Bcl-2, and Bax in Primary Breast Cancer Breast J 2000 6 44 52 10.1046/j.1524-4741.2000.98094.x 11348334

[B260] Baekelandt M Holm R Nesland JM Tropé CG Kristensen GB Expression of apoptosis-related proteins is an independent determinant of patient prognosis in advanced ovarian cancer J Clin Oncol 2000 18 3775 3781 11078490 10.1200/JCO.2000.18.22.3775

[B261] Yang M Yuan F Li P Chen Z Chen A Li S Hu C Interferon regulatory factor 4 binding protein is a novel p53 target gene and suppresses cisplatin-induced apoptosis of breast cancer cells Mol Cancer 2012 11 54 10.1186/1476-4598-11-54 22888789 PMC3447665

[B262] Perik PJ Van der Graaf WTA De Vries EGE Boomsma F Messerschmidt J Van Veldhuisen DJ Sleijfer DT Gietema JA Circulating apoptotic proteins are increased in long-term disease-free breast cancer survivors Acta Oncol 2006 45 175 183 10.1080/02841860500482225 16546863

[B263] Vejda S Posovszky C Zelzer S Peter B Bayer E Gelbmann D Schulte-Hermann R Gerner C Plasma from cancer patients featuring a characteristic protein composition mediates protection against apoptosis Mol Cell Proteomics 2002 1 387 393 10.1074/mcp.M200004-MCP200 12118080

[B264] Deryugina EI Quigley JP Matrix metalloproteinases and tumor metastasis Cancer Metastasis Rev 2006 25 9 34 10.1007/s10555-006-7886-9 16680569

[B265] Mangia A Malfettone A Rossi R Paradiso A Ranieri G Simone G Resta L Tissue remodelling in breast cancer: human mast cell tryptase as an initiator of myofibroblast differentiation Histopathology 2011 58 1096 1106 10.1111/j.1365-2559.2011.03842.x 21707711

[B266] Parashurama N Lobo NA Ito K Mosley AR Habte FG Zabala M Smith BR Lam J Weissman IL Clarke MF Gambhir SS Remodeling of endogenous mammary epithelium by breast cancer stem cells Stem Cells 2012 30 2114 2127 10.1002/stem.1205 22899386 PMC4158927

[B267] Kim BG Gao M-Q Choi YP Kang S Park HR Kang KS Cho NH Invasive breast cancer induces laminin-332 upregulation and integrin β4 neoexpression in myofibroblasts to confer an anoikis-resistant phenotype during tissue remodeling Breast Cancer Res Treat 2012 14 R88 10.1186/bcr3203 PMC3446351 22673183

[B268] Timmermans AM Montazeri H Trapman-Jansen AM Martens JW Foekens JA Umar A Abstract 806: Extracellular matrix metalloprotease inducer (EMMPRIN) and CD44 protein complexes are exclusively formed in basal- and normal-like breast cancer cell lines Cancer Res 2012 72 806 806 10.1158/1538-7445.AM2012-806

[B269] Glunde K Guggino SE Solaiyappan M Pathak AP Ichikawa Y Bhujwalla ZM Extracellular acidification alters lysosomal trafficking in human breast cancer cells Neoplasia 2003 5 533 545 14965446 10.1016/s1476-5586(03)80037-4 PMC1502575

[B270] Imai Y Ohmori K Yasuda S Wada M Suzuki T Fukuda K Ueda Y Breast cancer resistance protein/ABCG2 is differentially regulated downstream of extracellular signal-regulated kinase Cancer Sci 2009 100 1118 1127 10.1111/j.1349-7006.2009.01154.x 19514121 PMC11158436

[B271] Celis JE Moreira JMA Cabezón T Gromov P Friis E Rank F Gromova I Identification of extracellular and intracellular signaling components of the mammary adipose tissue and its interstitial fluid in high risk breast cancer patients: toward dissecting the molecular circuitry of epithelial-adipocyte stromal cell interactions Mol Cell Proteomics 2005 4 492 522 10.1074/mcp.M500030-MCP200 15695426

[B272] Cos S González A Martínez-Campa C Mediavilla MD Alonso-González C Sánchez-Barceló EJ Estrogen-signaling pathway: a link between breast cancer and melatonin oncostatic actions Cancer Detect Prev 2006 30 118 128 10.1016/j.cdp.2006.03.002 16647824

[B273] Malhotra GK Zhao X Band H Band V Shared signaling pathways in normal and breast cancer stem cells J Carcinog 2011 10 38 10.4103/1477-3163.91413 22279423 PMC3263309

[B274] Eroles P Bosch A Pérez-Fidalgo JA Lluch A Molecular biology in breast cancer: intrinsic subtypes and signaling pathways Cancer Treat Rev 2012 38 698 707 10.1016/j.ctrv.2011.11.005 22178455

[B275] Thomson CA Diet and breast cancer: understanding risks and benefits Nutr Clin Pract 2012 27 636 650 10.1177/0884533612454302 22948801

[B276] Giacosa A Barale R Bavaresco L Gatenby P Gerbi V Janssens J Johnston B Kas K La Vecchia C Mainguet P Morazzoni P Negri E Pelucchi C Pezzotti M Rondanelli M Cancer prevention in Europe: the Mediterranean diet as a protective choice Eur J Cancer Prev 2013 22 90 95 10.1097/CEJ.0b013e328354d2d7 22644232

[B277] Raouf A Sun Y Chatterjee S Basak P The biology of human breast epithelial progenitors Semin Cell Dev Biol 2012 23 606 612 10.1016/j.semcdb.2012.04.009 22609813

[B278] Pallavi R Giorgio M Pelicci PG Insights into the beneficial effect of caloric/ dietary restriction for a healthy and prolonged life Front Physiol 2012 3 318 22934068 10.3389/fphys.2012.00318 PMC3429088

[B279] Cole SW Chronic inflammation and breast cancer recurrence J Clin Oncol 2009 27 3418 3419 10.1200/JCO.2009.21.9782 19470918 PMC4828958

[B280] Early Breast Cancer Trialists' Collaborative Group (EBCTCG) Effects of chemotherapy and hormonal therapy for early breast cancer on recurrence and 15-year survival: an overview of the randomised trials Lancet 2005 365 1687 1717 15894097 10.1016/S0140-6736(05)66544-0

[B281] Slamon DJ Clark GM Wong SG Levin WJ Ullrich A McGuire WL Human breast cancer: correlation of relapse and survival with amplification of the HER-2/neu oncogene Science 1987 235 177 182 10.1126/science.3798106 3798106

[B282] Ross JS Slodkowska EA Symmans WF Pusztai L Ravdin PM Hortobagyi GN The HER-2 receptor and breast cancer: ten years of targeted anti-HER-2 therapy and personalized medicine Oncologist 2009 14 320 368 10.1634/theoncologist.2008-0230 19346299

[B283] De Laurentiis M Arpino G Massarelli E Ruggiero A Carlomagno C Ciardiello F Tortora G D’Agostino D Caputo F Cancello G Montagna E Malorni L Zinno L Lauria R Bianco AR De Placido S A meta-analysis on the interaction between HER-2 expression and response to endocrine treatment in advanced breast cancer Clin Cancer Res 2005 11 4741 4748 10.1158/1078-0432.CCR-04-2569 16000569

[B284] Hayes DF Thor AD Dressler LG Weaver D Edgerton S Cowan D Broadwater G Goldstein LJ Martino S Ingle JN Henderson IC Norton L Winer EP Hudis CA Ellis MJ Berry DA HER2 and response to paclitaxel in node-positive breast cancer N Engl J Med 2007 357 1496 1506 10.1056/NEJMoa071167 17928597

[B285] Pritchard KI Shepherd LE O’Malley FP Andrulis IL Tu D Bramwell VH Levine MN HER2 and responsiveness of breast cancer to adjuvant chemotherapy N Engl J Med 2006 354 2103 2111 10.1056/NEJMoa054504 16707747

[B286] Gianni L Pienkowski T Im Y-H Roman L Tseng L-M Liu M-C Lluch A Staroslawska E de la Haba-Rodriguez J Im S-A Pedrini JL Poirier B Morandi P Semiglazov V Srimuninnimit V Bianchi G Szado T Ratnayake J Ross G Valagussa P Efficacy and safety of neoadjuvant pertuzumab and trastuzumab in women with locally advanced, inflammatory, or early HER2-positive breast cancer (NeoSphere): a randomised multicentre, open-label, phase 2 trial Lancet Oncol 2012 13 25 32 10.1016/S1470-2045(11)70336-9 22153890

[B287] Slamon DJ Leyland-Jones B Shak S Fuchs H Paton V Bajamonde A Fleming T Eiermann W Wolter J Pegram M Baselga J Norton L Use of chemotherapy plus a monoclonal antibody against HER2 for metastatic breast cancer that overexpresses HER2 N Engl J Med 2001 344 783 792 10.1056/NEJM200103153441101 11248153

[B288] Geyer CE Forster J Lindquist D Chan S Romieu CG Pienkowski T Jagiello-Gruszfeld A Crown J Chan A Kaufman B Skarlos D Campone M Davidson N Berger M Oliva C Rubin SD Stein S Cameron D Lapatinib plus capecitabine for HER2-positive advanced breast cancer N Engl J Med 2006 355 2733 2743 10.1056/NEJMoa064320 17192538

[B289] Urruticoechea A Smith IE Dowsett M Proliferation marker Ki-67 in early breast cancer J Clin Oncol 2005 23 7212 7220 10.1200/JCO.2005.07.501 16192605

[B290] Yerushalmi R Woods R Ravdin PM Hayes MM Gelmon KA Ki67 in breast cancer: prognostic and predictive potential Lancet Oncol 2010 11 174 183 10.1016/S1470-2045(09)70262-1 20152769

[B291] Chang J Powles TJ Allred DC Ashley SE Makris A Gregory RK Osborne CK Dowsett M Prediction of clinical outcome from primary tamoxifen by expression of biologic markers in breast cancer patients Clin Cancer Res 2000 6 616 621 10690547

[B292] Fasching PA Heusinger K Haeberle L Niklos M Hein A Bayer CM Rauh C Schulz-Wendtland R Bani MR Schrauder M Kahmann L Lux MP Strehl JD Hartmann A Dimmler A Beckmann MW Wachter DL Ki67, chemotherapy response, and prognosis in breast cancer patients receiving neoadjuvant treatment BMC Cancer 2011 11 486 10.1186/1471-2407-11-486 22081974 PMC3262864

[B293] Goldhirsch A Wood WC Coates AS Gelber RD Thürlimann B Senn H-J Strategies for subtypes--dealing with the diversity of breast cancer: highlights of the St. Gallen International Expert Consensus on the Primary Therapy of Early Breast Cancer 2011 Ann Oncol 2011 22 1736 1747 10.1093/annonc/mdr304 21709140 PMC3144634

[B294] Harbeck N Dettmar P Thomssen C Henselmann B Kuhn W Ulm K Jänicke F Höfler H Graeff H Schmitt M Prognostic impact of tumor biological factors on survival in node-negative breast cancer Anticancer Res 1998 18 2187 2197 9703782

[B295] Jänicke F Schmitt M Pache L Ulm K Harbeck N Höfler H Graeff H Urokinase (uPA) and its inhibitor PAI-1 are strong and independent prognostic factors in node-negative breast cancer Breast Cancer Res Treat 1993 24 195 208 10.1007/BF01833260 8435475

[B296] Annecke K Schmitt M Euler U Zerm M Paepke D Paepke S von Minckwitz G Thomssen C Harbeck N uPA and PAI-1 in breast cancer: review of their clinical utility and current validation in the prospective NNBC-3 trial Adv Clin Chem 2008 45 31 45 18429492 10.1016/s0065-2423(07)00002-9

[B297] Jänicke F Prechtl A Thomssen C Harbeck N Meisner C Untch M Sweep CG Selbmann HK Graeff H Schmitt M Randomized adjuvant chemotherapy trial in high-risk, lymph node-negative breast cancer patients identified by urokinase-type plasminogen activator and plasminogen activator inhibitor type 1 J Natl Cancer Inst 2001 93 913 920 10.1093/jnci/93.12.913 11416112

[B298] Adjuvant! Online[http://www.adjuvantonline.com/index.jsp]

[B299] Mook S Schmidt MK Rutgers EJ van de Velde AO Visser O Rutgers SM Armstrong N Van’t Veer LJ Ravdin PM Calibration and discriminatory accuracy of prognosis calculation for breast cancer with the online Adjuvant! program: a hospital-based retrospective cohort study Lancet Oncol 2009 10 1070 1076 10.1016/S1470-2045(09)70254-2 19801202

[B300] Olivotto IA Bajdik CD Ravdin PM Speers CH Coldman AJ Norris BD Davis GJ Chia SK Gelmon KA Population-based validation of the prognostic model ADJUVANT! for early breast cancer J Clin Oncol 2005 23 2716 2725 15837986 10.1200/JCO.2005.06.178

[B301] Ozanne EM Braithwaite D Sepucha K Moore D Esserman L Belkora J Sensitivity to input variability of the Adjuvant! Online breast cancer prognostic model J Clin Oncol 2009 27 214 219 10.1200/JCO.2008.17.3914 19047286

[B302] Eastern Cancer Registry and Information Centre PREDICT[http://www.predict.nhs.uk/]

[B303] Wishart GC Bajdik CD Azzato EM Dicks E Greenberg DC Rashbass J Caldas C Pharoah PDP A population-based validation of the prognostic model PREDICT for early breast cancer Eur J Surg Oncol 2011 37 411 417 10.1016/j.ejso.2011.02.001 21371853

[B304] Wishart GC Bajdik CD Dicks E Provenzano E Schmidt MK Sherman M Greenberg DC Green AR Gelmon KA Kosma V-M Olson JE Beckmann MW Winqvist R Cross SS Severi G Huntsman D Pylkäs K Ellis I Nielsen TO Giles G Blomqvist C Fasching PA Couch FJ Rakha E Foulkes WD Blows FM Bégin LR Van’t Veer LJ Southey M Nevanlinna H Mannermaa A Cox A Cheang M Baglietto L Caldas C Garcia-Closas M Pharoah PDP PREDICT Plus: development and validation of a prognostic model for early breast cancer that includes HER2 Br J Cancer 2012 107 800 807 10.1038/bjc.2012.338 22850554 PMC3425970

[B305] Cheang MCU van de Rijn M Nielsen TO Gene expression profiling of breast cancer Annu Rev Pathol 2008 3 67 97 10.1146/annurev.pathmechdis.3.121806.151505 18039137

[B306] Perou CM Sørlie T Eisen MB van de Rijn M Jeffrey SS Rees CA Pollack JR Ross DT Johnsen H Akslen LA Fluge O Pergamenschikov A Williams C Zhu SX Lønning PE Børresen-Dale AL Brown PO Botstein D Molecular portraits of human breast tumours Nature 2000 406 747 752 10.1038/35021093 10963602

[B307] Rouzier R Perou CM Symmans WF Ibrahim N Cristofanilli M Anderson K Hess KR Stec J Ayers M Wagner P Morandi P Fan C Rabiul I Ross JS Hortobagyi GN Pusztai L Breast cancer molecular subtypes respond differently to preoperative chemotherapy Clin Cancer Res 2005 11 5678 5685 10.1158/1078-0432.CCR-04-2421 16115903

[B308] Smid M Wang Y Zhang Y Sieuwerts AM Yu J Klijn JGM Foekens JA Martens JWM Subtypes of breast cancer show preferential site of relapse Cancer Res 2008 68 3108 3114 10.1158/0008-5472.CAN-07-5644 18451135

[B309] Sørlie T Perou CM Tibshirani R Aas T Geisler S Johnsen H Hastie T Eisen MB van de Rijn M Jeffrey SS Thorsen T Quist H Matese JC Brown PO Botstein D Lønning PE Børresen-Dale AL Gene expression patterns of breast carcinomas distinguish tumor subclasses with clinical implications Proc Natl Acad Sci U S A 2001 98 10869 10874 10.1073/pnas.191367098 11553815 PMC58566

[B310] Paik S Shak S Tang G Kim C Baker J Cronin M Baehner FL Walker MG Watson D Park T Hiller W Fisher ER Wickerham DL Bryant J Wolmark N A multigene assay to predict recurrence of tamoxifen-treated, node-negative breast cancer N Engl J Med 2004 351 2817 2826 10.1056/NEJMoa041588 15591335

[B311] Veer LJV’t Dai H van de Vijver MJ He YD Hart AAM Mao M Peterse HL van der Kooy K Marton MJ Witteveen AT Schreiber GJ Kerkhoven RM Roberts C Linsley PS Bernards R Friend SH Gene expression profiling predicts clinical outcome of breast cancer Nature 2002 415 530 536 10.1038/415530a 11823860

[B312] Buyse M Loi S Van’t Veer L Viale G Delorenzi M Glas AM D’ Assignies MS Bergh J Lidereau R Ellis P Harris A Bogaerts J Therasse P Floore A Amakrane M Piette F Rutgers E Sotiriou C Cardoso F Piccart MJ Validation and clinical utility of a 70-gene prognostic signature for women with node-negative breast cancer J Natl Cancer Inst 2006 98 1183 1192 10.1093/jnci/djj329 16954471

[B313] van de Vijver MJ He YD Veer LJ V Dai H Hart AAM Voskuil DW Schreiber GJ Peterse JL Roberts C Marton MJ Parrish M Atsma D Witteveen A Glas A Delahaye L van der Velde T Bartelink H Rodenhuis S Rutgers ET Friend SH Bernards R A gene-expression signature as a predictor of survival in breast cancer N Engl J Med 2002 347 1999 2009 10.1056/NEJMoa021967 12490681

[B314] Straver ME Glas AM Hannemann J Wesseling J van de Vijver MJ Rutgers EJT Vrancken Peeters M-JTFD van Tinteren H Van’t Veer LJ Rodenhuis S The 70-gene signature as a response predictor for neoadjuvant chemotherapy in breast cancer Breast Cancer Res Treat 2010 119 551 558 10.1007/s10549-009-0333-1 19214742

[B315] Paik S Tang G Shak S Kim C Baker J Kim W Cronin M Baehner FL Watson D Bryant J Costantino JP Geyer CE Jr Wickerham DL Wolmark N Gene expression and benefit of chemotherapy in women with node-negative, estrogen receptor-positive breast cancer J Clin Oncol 2006 24 3726 3734 10.1200/JCO.2005.04.7985 16720680

[B316] Albain KS Barlow WE Shak S Hortobagyi GN Livingston RB Yeh I-T Ravdin P Bugarini R Baehner FL Davidson NE Sledge GW Winer EP Hudis C Ingle JN Perez EA Pritchard KI Shepherd L Gralow JR Yoshizawa C Allred DC Osborne CK Hayes DF Prognostic and predictive value of the 21-gene recurrence score assay in postmenopausal women with node-positive, oestrogen-receptor-positive breast cancer on chemotherapy: a retrospective analysis of a randomised trial Lancet Oncol 2010 11 55 65 10.1016/S1470-2045(09)70314-6 20005174 PMC3058239

[B317] Rutgers E Piccart-Gebhart MJ Bogaerts J Delaloge S Veer LVT Rubio IT Viale G Thompson AM Passalacqua R Nitz U Vindevoghel A Pierga J-Y Ravdin PM Werutsky G Cardoso F The EORTC 10041/BIG 03–04 MINDACT trial is feasible: results of the pilot phase Eur J Cancer 2011 47 2742 2749 10.1016/j.ejca.2011.09.016 22051734

[B318] Sparano JA Paik S Development of the 21-gene assay and its application in clinical practice and clinical trials J Clin Oncol 2008 26 721 728 10.1200/JCO.2007.15.1068 18258979

